# Advancing food preservation with quercetin-based Nanocomposites: Antimicrobial, antioxidant, and controlled-release strategies - A review

**DOI:** 10.1016/j.crfs.2025.101159

**Published:** 2025-08-09

**Authors:** Sakshi Jasrotia, Sonali Gupta, Manas Laxman Kudipady, Yashoda Malgar Puttaiahgowda

**Affiliations:** a Department of Chemistry, Manipal Institute of Technology, Manipal Academy of Higher Education, Manipal, Karnataka, 576104, India; b Department of Information and Communication Technology, Manipal Institute of Technology, Manipal Academy of Higher Education, Manipal, Karnataka, 576104, India

**Keywords:** Flavonoid encapsulation, Biopolymer matrices, Shelf-life extension, Electrospinning, Pathogen inhibition

## Abstract

The global food waste crisis, driven by rapid spoilage and oxidative degradation, substantiates the urgent need for sustainable packaging innovations. Though mechanically robust, conventional petroleum-based polymers contribute significantly to environmental pollution due to their non-biodegradability. Quercetin (Qr), a bioactive flavonoid with potent antimicrobial and antioxidant properties, has emerged as a promising component in next-generation active packaging. This review examines Qr-functionalized nanocomposite films, focusing on their ability to inhibit pathogens (e.g., *Escherichia coli, Salmonella, Staphylococcus aureus*) and prevent oxidative damage through radical scavenging and metal chelation. Advanced fabrication methods like solution casting and electrospinning enhance Qr's controlled release, mechanical strength, and UV-shielding capabilities. The synergistic use of biopolymers (e.g., CS, PVA) and nanofillers (e.g., ZnO, Ag NPs) further enhances thermal stability, biodegradability, and antimicrobial performance. Studies show that Qr-based films can extend food shelf life by up to 50 %, significantly reducing microbial loads and oxidative markers. Despite its GRAS status, challenges like production scalability, cost-effectiveness, and long-term stability remain. By integrating nanotechnology with bioactive compounds like Qr, this innovation represents a paradigm shift toward intelligent, eco-friendly packaging aligned with the United Nations Sustainable Development Goals (SDGs).

## Highlights

• Quercetin, a natural flavonoid, exhibits strong antioxidant and antimicrobial properties.
• Polymer-based quercetin composites enhance stability and controlled release for biomedical and packaging applications.
• Functionalized quercetin films show potential in food preservation and wound healing.
• Synergistic effects with nanoparticles improve quercetin's bioavailability and antimicrobial efficiency.
• Future research focuses on optimizing quercetin-polymer interactions for sustainable and high-performance applications.

## Graphical abstract

**Figure:**
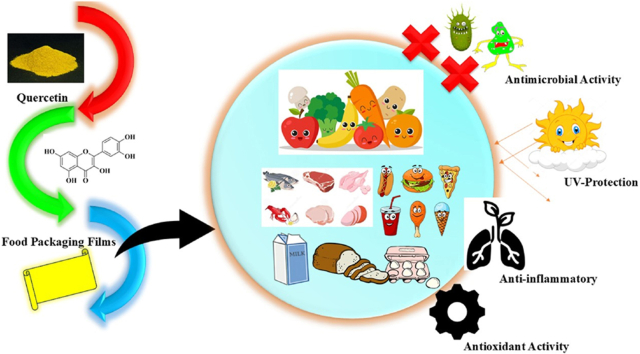

## Abbreviations:

**Quercetin**: Qr
**PVA**: Polyvinyl alcohol
**DPPH**: 2,2-diphenyl-1-picrylhydrazyl
**ABTS**: Azino-bis(3-ethylbenzothiazoline-6-sulfonic acid
**TS**: Tensile strength
**EAB**: Elongation at break
**EM**: Elastic modulus
**YM**: Young's modulus
**WVP**: Water vapor permeability
**WVTR**: Water vapor transmission rate
**WCA**: Water contact angle
**WS**: Water solubility
**HOMO**: Highest occupied molecular orbital
**ROS**: Reactive oxygen species
**PBAT**: Poly(butylene adipate-co-terephthalate
**LDH**: Layered double hydroxide
**NIR**: Near-infrared radiation
**TPS**: Thermoplastic starch
**OMMT**: Modified montmorillonite
**CS**: Chitosan
**KGM**: Konjac glucomannan
**PCL:** Polycaprolactone
**β-CD**: β-cyclodextrin
**TG**: Tragacanth gum
**CMCS**: Carboxymethyl chitosan
**NE**: Nanoemulsions
**QNLs**: Quercetin-loaded nanoliposomes
**TBARs**: Thiobarbituric acid reactive substances
**TVB-N:** Total volatile basic nitrogen
**CG**: κ-carrageenan
**ELE**: Eucalyptus leaf extract
**PVC**: Poly(vinyl chloride)
**RO**: Rosemary essential oil
**DES**: Deep eutectic solvent
**MC**: Methylcellulose
**CNFs**: Nanofibers
**NAT**: Natamycin
**CNC**: Cellulose nanocrystals
**PBS**: Poly(butylene succinate)
**ZnO**: Zinc oxide
**AgNPs**: Silver nanoparticles
**GO**: Graphene oxide
**NPs**: Nanoparticles

## 1 Introduction

Food waste constitutes a substantial global challenge, with approximately 1.3 billion metric tons generated annually, leading to economic losses quantified in the hundreds of billions of dollars (Blakeney, 2019), (FAO, 2013). This highly biodegradable waste undergoes rapid decomposition, emitting malodorous volatiles and greenhouse gases while fostering pathogen growth, exacerbating environmental and public health risks (Pan et al., 2024a). Food loss predominantly occurs during the production and distribution phases, whereas food waste is primarily observed at the consumer level, as well as within the retail and food service sectors (Brennan and Browne, 2021). Determining the shelf-life of packaged food, and the period it maintains acceptable quality, is a key waste reduction strategy, contingent on the product's intrinsic properties and packaging (Valero et al., 2012). Traditional packaging primarily functions as a passive barrier against external factors like oxygen, moisture, and light, slowing deterioration when well-designed. Unlike active packaging, however, it does not interact with the product or its environment to actively preserve quality or extend shelf life (Park et al., 2022), (Qiu et al., 2020). To combat the limitations of traditional packaging in preventing spoilage and mitigating escalating food waste, antimicrobial packaging is emerging as a promising and strategic intervention. Antimicrobial packaging, incorporating materials embedded with natural antimicrobial agents, represents an innovative approach to suppressing microbial proliferation in food products. The packaging industry has demonstrated growing interest in these solutions due to their demonstrated efficacy in enhancing food quality, safety, and extending shelf life (Upadhyay et al., 2024). Several techniques are employed for the synthesis of polymeric films, including solvent casting, tape-casting, compression molding, injection molding, as well as various extrusion methods such as single-screw, twin-screw, reactive, co-extrusion, and blown-extrusion (do Val Siqueira et al., 2021). Among natural bioactive compounds, Qr has emerged as a highly promising agent due to its potent antioxidant and antimicrobial properties, positioning it as an ideal component for incorporation into advanced, next-generation packaging systems (Arias et al., 2025) (see Table 1, Table 2).

**Table 1:** Comparative analysis of bioactive compounds in advanced food packaging systems.

Compound	Source	Benefits	References
Phenolic Compounds	Fruits, vegetables, herbs, spices, tea, chocolate, wine	Antioxidant and antibacterial properties improve sensory attributes, protect against oxidation	(Singh et al., 2022)
Quercetin	Onions, red wine, olive oil, berries, and grapefruit	Strong antioxidant and anti-inflammatory effects, prevent blood clotting	(López-de-Dicastillo et al., 2010)
Hesperidin	Citrus fruits, mushrooms	Antioxidant, anti-inflammatory, antimicrobial, and anticancer effects	(Pan et al., 2024b)
Clove Oil	Clove	Antimicrobial and antioxidant properties retards food spoilage	(Chandran et al., 2024)
Argan Oil	Argan	Antimicrobial and antioxidant properties, UV protection	Tang et al. (2025)
Rutin	Buckwheat, red pepper, tomato skin	Anticancer, antioxidant, and cytoprotective properties	(Ho, 1992)
Citrus Peel Extracts	Citrus fruits	Antibacterial, antifungal, antioxidant, and anti-inflammatory properties due to flavonoids, carotenoids, and phenolics	(Deshmukh and Gaikwad, 2024)
trans-2-hexenal	Strawberries	Antifungal properties inhibit mold growth	(Chandran et al., 2024)
Catechin	Green tea, apples	Improves thermal resistance of packaging films and antioxidant agent	(López-de-Dicastillo et al., 2010)
Kaempferol	Kale, spinach, broccoli	Antioxidant and anti-inflammatory properties; potential to inhibit cancer cell growth	(Ho, 1992)
Apigenin	Parsley, chamomile	Exhibits anti-inflammatory and antioxidant effects; may enhance the quality of food preservation	(Chandran et al., 2024)
Luteolin	Celery, green pepper	Known for its antioxidant properties and ability to inhibit certain bacteria	(Ho, 1992)
Myricetin	Berries, grapes	Exhibits strong antioxidant activity; can protect against oxidative stress	(Tang et al., 2025)
Naringenin	Grapefruit	Anti-inflammatory effects; potential to improve the sensory attributes of food products	(Pan et al., 2024b)
Tannic Acid (Tannins)	Various plants (e.g., tea leaves, oak bark)	Provides antioxidant and antimicrobial properties; improves mechanical strength and barrier properties in packaging	(Singh et al., 2022), (López-de-Dicastillo et al., 2010)

**Table 2:** Description of various film nanocomposite fabrication techniques.

Film Nanocomposite	Fabrication Technique	Description	References
Quercetin-Cellulose Acetate-AgNPs	Solution Casting	Antimicrobial food packaging with AgNPs for bacterial inhibition	(Marrez et al., 2018)
Quercetin-CS-Gelatin	Solvent Evaporation	Improved TS, UV blocking, and antimicrobial activity	(Yadav et al., 2020)
Quercetin-CS Nanoparticle Film	Electrospinning	Nanocomposite films for enhanced mechanical and antimicrobial properties	(Ezati and Rhim, 2021)
Quercetin-PLA/Nanocellulose	Electrospinning	Multilayer active packaging with improved barrier properties	(López de Dicastillo et al., 2021)
Quercetin-OMMT/PBAT/TPS	Nanoparticle Incorporation	Enhanced oxygen barrier, UV shielding, and biodegradability	(Yang et al., 2023)
Quercetin-ZnO-PVA Film	Crosslinking with Genipin/Epoxy	Multifunctional film with improved antimicrobial properties	(Sani et al., 2023)
Quercetin-AgNPs-GO	Thermal Treatment/UV Curing	Nanocomposite film for extended durability and antioxidant activity	(Yin et al., 2024)
Quercetin-Functionalized Layered Clay/PVA/CS	Solution Casting	Enhanced mechanical, barrier, and antimicrobial properties	(Wang et al., 2024)
Quercetin-Starch Nanoparticle Film	LDH and Starch NPs	Controlled release system for extended food preservation	(Zeng et al., 2024)
Quercetin-Wheat Gluten/ZnO Nanoliposome	NIR	Antimicrobial film for seafood preservation	(Bakeshlou et al., 2024a)
Quercetin-β-Cyclodextrin/TG/CMCS	Solution Casting	Bio-based food packaging with high antioxidant activity	(Liu et al., 2025)
Quercetin-Zein-Gelatin Nanoparticle Film	Electrospinning	High barrier properties and extended strawberry preservation	(Xu et al., 2025)

Quercetin (3,3′,4′5,7-pentahydroxyflavone), a benzo-r-pyrone derivative, is a bioactive flavonoid ubiquitously distributed across diverse plant species (Formica and Regelson, 1995). It can scavenge ROS, such as hydroxyl radicals and superoxide anions, thereby mitigating oxidative stress. This intrinsic property underpins its wide spectrum of biological and pharmacological activities, including potent anti-inflammatory, anti-allergic, antioxidant, and antimicrobial effects (Li et al., 2016), (Yilmaz et al., 2014). However, during processing and storage, Qr is susceptible to degradation due to factors such as ambient pH, temperature, and humidity, which diminish its biological activity and lead to a loss of its beneficial properties (Yin et al., 2024). To address these limitations, nanotechnology has emerged as a transformative tool, enabling the stabilization and controlled release of bioactive compounds like Qr in food packaging applications (Weiss et al., 2006).

Recent breakthroughs in nanotechnology have revolutionized the field of food packaging by offering innovative solutions to enhance the functionality and performance of packaging materials (Pathade, 2024). Nanocomposite films, which integrate nanoscale fillers within polymer matrices, have garnered considerable interest owing to their enhanced mechanical strength, improved barrier capabilities, and potent antimicrobial characteristics. These advanced materials can be precisely designed to facilitate the controlled release of bioactive agents, thereby maintaining prolonged antimicrobial and antioxidant efficacy over the entire shelf life of packaged food products (Maity et al., 2023). The integration of Qr into nanocomposite films represents a promising strategy to leverage its bioactive properties while overcoming its inherent instability (Bakr et al., 2024). Integrating Qr into nanocomposite films represents a promising strategy to leverage its bioactive potential while addressing its intrinsic instability. Through encapsulation within nanocarriers or dispersion in nanofiller-enhanced polymer matrices, the bioavailability and functional efficacy of Qr can be markedly improved, positioning it as a viable component for advanced active food packaging systems (Sun et al., 2025). In this review, we explore recent advancements in Qr-based nanocomposite films for food packaging, focusing on their enhanced mechanical, barrier, and antimicrobial properties. We discuss the integration of Qr into various polymer matrices and nanocarriers, emphasizing its role in improving food safety, extending shelf life, and reducing food waste. Additionally, we address challenges such as scalability, stability, and real-world performance, while outlining future research directions to optimize these materials for commercial use. This review highlights the potential of Qr-functionalized nanomaterials and underscores the need for further research into their practical applications and environmental impact.

## 2 Natural occurrences, structural chemistry, and properties of quercetin

Qr, a prominent flavonol within the flavonoid subgroups of polyphenols, is ubiquitously distributed in various plant-based foods, including fruits, vegetables, leaves, seeds, and grains, with an average daily intake of 25–50 mg in human diets (Baqer et al., 2024). Structurally, Qr is defined as 2-(3,5,7,3′,4′-pentahydroxyflavone), comprising three aromatic benzene rings and five hydroxyl groups (Aghababaei and Hadidi, 2023). These hydroxyl groups are critical determinants of its biological activity, including its potent antioxidant properties. Qr exists in two primary forms: as an aglycone, which lacks a carbohydrate moiety, and as a glycoside, where hydroxyl groups are substituted with sugar residues. The glycosylation of Qr significantly alters its physicochemical properties and bioavailability (Tang et al., 2025). The biological activities of Qr are largely attributed to its antioxidant capacity, which arises from its ability to scavenge free radicals and chelate metal ions, rendering it a subject of extensive research in chronic diseases and metabolic disorders (Nyarko, 2024), (Kerna et al., 2024).

Incorporating Qr into food packaging films enhances their mechanical, thermal, antioxidant, antimicrobial, and barrier properties while contributing to biodegradability. Qr improves the mechanical strength of films, exhibiting increased YM and EAB, and influences thermal properties by shifting glass transition and crystallization temperatures to higher values, thereby improving thermal stability (Olewnik-Kruszkowska et al., 2021). Its strong antioxidant activity helps prevent oxidative degradation of food products by effectively scavenging free radicals (Łopusiewicz et al., 2021), (Shabir et al., 2022). Moreover, Qr demonstrates significant antimicrobial properties against pathogens like *Escherichia coli* and *Staphylococcus aureus* (Najim et al., 2024), and provides UV-blocking capabilities for light-sensitive products (Lee et al., 2024) and supports biodegradable packaging development, aligning with sustainable packaging solutions (Guo et al., 2024).

### 2.1 Mechanism of action

**Fig. 1:**
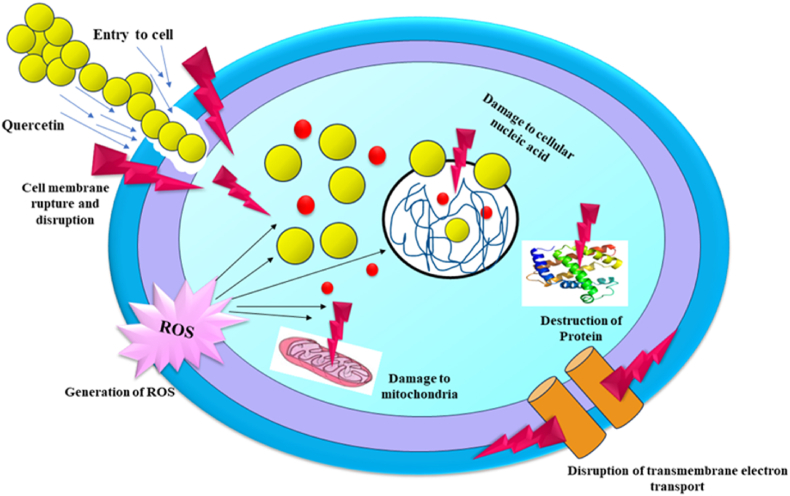
Pictorial representation of antimicrobial mechanisms exhibited by Quercetin.

Qr exerts its antimicrobial and antioxidant effects through a variety of mechanisms. Its antimicrobial activity stems from its ability to damage bacterial cell membranes, leading to increased permeability and cell death (Nguyen and Bhattacharya, 2022). This disruption is attributed to Qr's interaction with the lipid bilayer, causing structural disorganization. Gram-positive bacteria are generally more susceptible, while Gram-negative bacteria exhibit greater resistance due to differences in membrane composition. Qr also inhibits the synthesis of nucleic acids and proteins, essential for bacterial survival and replication. It disrupts biofilm formation by interfering with bacterial quorum sensing, preventing adhesion and biofilm production in species such as *Serratia marcescens*, *Streptococcus mutans*, and drug-resistant *Staphylococcus aureus* (Kannan et al., 2022). Furthermore, Qr enhances the effectiveness of antibiotics by inhibiting bacterial efflux pumps, which normally expel toxic substances, thereby increasing intracellular antibiotic concentrations. It can also induce mitochondrial dysfunction, disrupting energy production and metabolic processes in bacterial cells. On the antioxidant front, Qr's chemical structure, particularly its hydroxyl groups, enables it to effectively scavenge free radicals, neutralizing their harmful effects. It also inhibits oxidative enzymes like lipoxygenases and xanthine oxidase, reducing the production of ROS (Fig. 1). Additionally, Qr chelates metal ions such as iron and copper, preventing them from catalyzing free radical formation. These multifaceted mechanisms highlight Qr's potential as a powerful antimicrobial and antioxidant agent, contributing to its utility in enhancing food safety, extending shelf life, and improving the functionality of packaging materials (Qi et al., 2022).

## 3 Comparative analysis of quercetin with other flavonoids used in food packaging

Qr demonstrates potent antibacterial and antifungal properties through multiple mechanisms, including disruption of bacterial cell membranes, inhibition of nucleic acid synthesis, and suppression of enzymatic activity. Its efficacy in antimicrobial food packaging surpasses that of other flavonoids, such as catechin and rutin, due to its unique ability to permeabilize bacterial membranes and inhibit biofilm formation (Baqer et al., 2024). Furthermore, Qr stands out as one of the most effective natural antioxidants, attributed to its distinctive molecular structure featuring five hydroxyl groups and a conjugated system that facilitates electron delocalization. This structural configuration endows Qr with a higher reaction rate with free radicals and a lower activation energy for DPPH reduction than catechin and naringenin (Veiko et al., 2021). The high HOMO energy and optimal orbital delocalization further enhance its antioxidant efficiency, making it effective for oxidative stress mitigation. In addition to its bioactive properties, Qr significantly improves the mechanical performance of biodegradable polymer films used in food packaging. Films incorporating Qr exhibit enhanced TS and flexibility, thereby increasing their resistance to physical stress and extending their functional durability. This flavonoid also synergistically enhances the antimicrobial activity of other bioactive compounds by increasing bacterial membrane permeability, as evidenced by studies showing amplified antibacterial effects when Qr is combined with other natural preservatives (Carrillo-Martinez et al., 2024). Such synergistic interactions make Qr an ideal component for active food packaging systems (Baqer et al., 2024). Moreover, Qr-based films maintain remarkable stability under typical food storage conditions while retaining biodegradability. Unlike other flavonoids, Qr demonstrates prolonged efficacy without significant degradation, ensuring sustained antimicrobial and antioxidant effects over extended periods (Baqer et al., 2024). These multifaceted advantages position Qr as a highly promising compound for advancing the development of functional, sustainable, and effective food packaging materials.

## 4 Fabrication techniques of quercetin-based nanocomposite film

The synthesis of Qr-based nanocomposite films in the reviewed paper was conducted using various techniques, including solution casting, electrospinning, solvent evaporation, and nanoparticle incorporation. The films were primarily fabricated using biopolymer matrices such as PVA, CS, gelatin, and PBAT, with Qr introduced either in its pure form or encapsulated within nanocarriers like zein NPs, β-cyclodextrin complexes, and metal-organic frameworks. Crosslinking agents such as genipin and epoxy chain extenders enhanced mechanical stability and controlled Qr release. Nanofillers such as ZnO, AgNPs, and GO were incorporated to improve antimicrobial efficacy and barrier properties. The synthesis processes often involve the dispersion of Qr within polymer solutions, followed by film formation through solvent evaporation, thermal treatment, or UV-curing. Additionally, LDH and carboxymethyl starch NPs were used for controlled Qr release, while NIR irradiation was explored for enhancing antimicrobial effects. The optimized films exhibited enhanced mechanical strength, barrier properties, antioxidant activity, and prolonged antimicrobial effectiveness, making them suitable for food packaging applications.

## 5 Advanced applications of quercetin-based nanocomposite films in food packaging

### 5.1 Packaging of fruits and vegetables

**Fig. 2:**
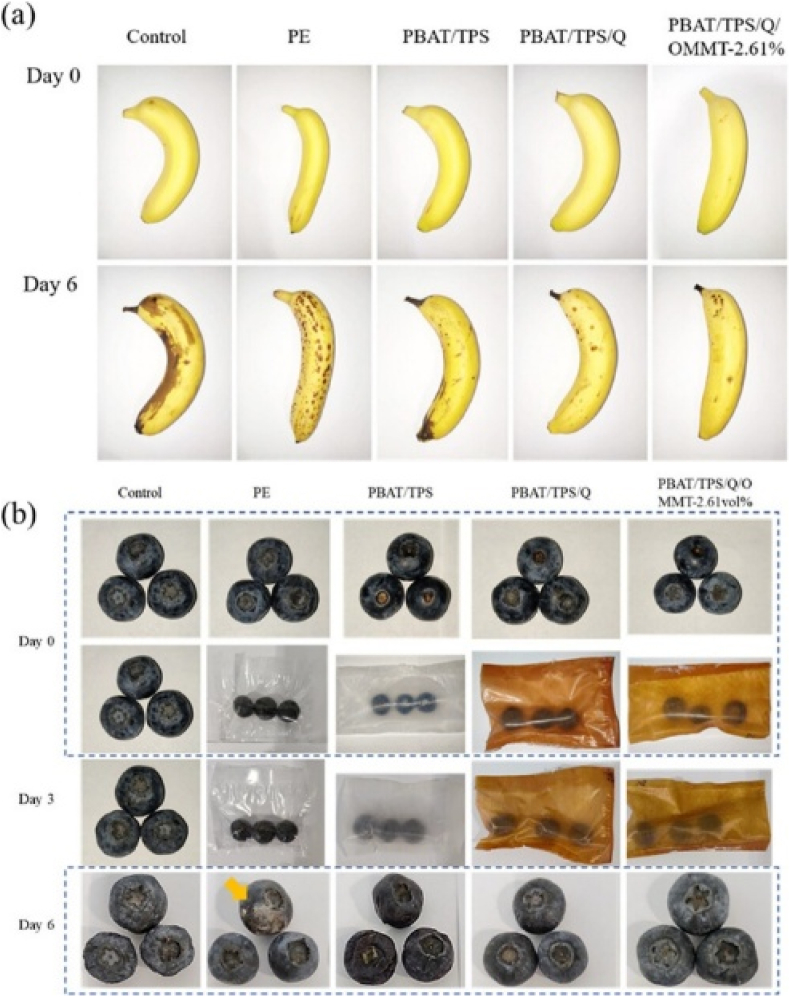
**Illustrates the appearance of (a) bananas and (b) blueberries during storage in various packaging films. Reprinted with permission from ref**(Yang et al., 2023). **copywriter (2023) ACS**.

In 2023, Yang et al. developed PBAT/TPS films incorporating Qr and organically modified OMMT for active food packaging. Fabrication involved pellet formation using counter-rotating twin-screw extrusion, followed by film preparation with a single-screw film-blowing machine (Kechuang LSJ20, China), which simulates industrial techniques but remains limited to laboratory-scale, batch-wise processing. Adding 3.34 vol% OMMT significantly improved the mechanical properties, increasing TS from 8.0 MPa to 11.5 MPa and EAB to 569.8 %. Barrier performance was notably enhanced, with oxygen permeability reduced by 87 % (to 0.13 Barrer at 2.61 vol% OMMT) and WVP decreased by 54 % (4.42 × 10^−13^ g cm/cm^2^·s·Pa). The films maintained thermal stability up to 200 °C without degradation. Functional properties were also enhanced. Antioxidant activity was strong, requiring just 0.03 g of material to quench 50 % of DPPH radicals. UV transmittance dropped by 50 % due to the combined effects of Qr's phenolic chromophores and OMMT's light-blocking ability. Biodegradability was improved by including starch, which served as a nutrient source for microbial degradation. The films effectively extended the shelf life of bananas and blueberries, maintaining freshness for 6 days and outperforming the control samples (Fig. 2). The study concludes that the films have strong commercial potential, although improvements in moisture resistance and scalable, continuous production methods are needed to compete with conventional LDPE packaging (Yang et al., 2023).

**Fig. 3:**
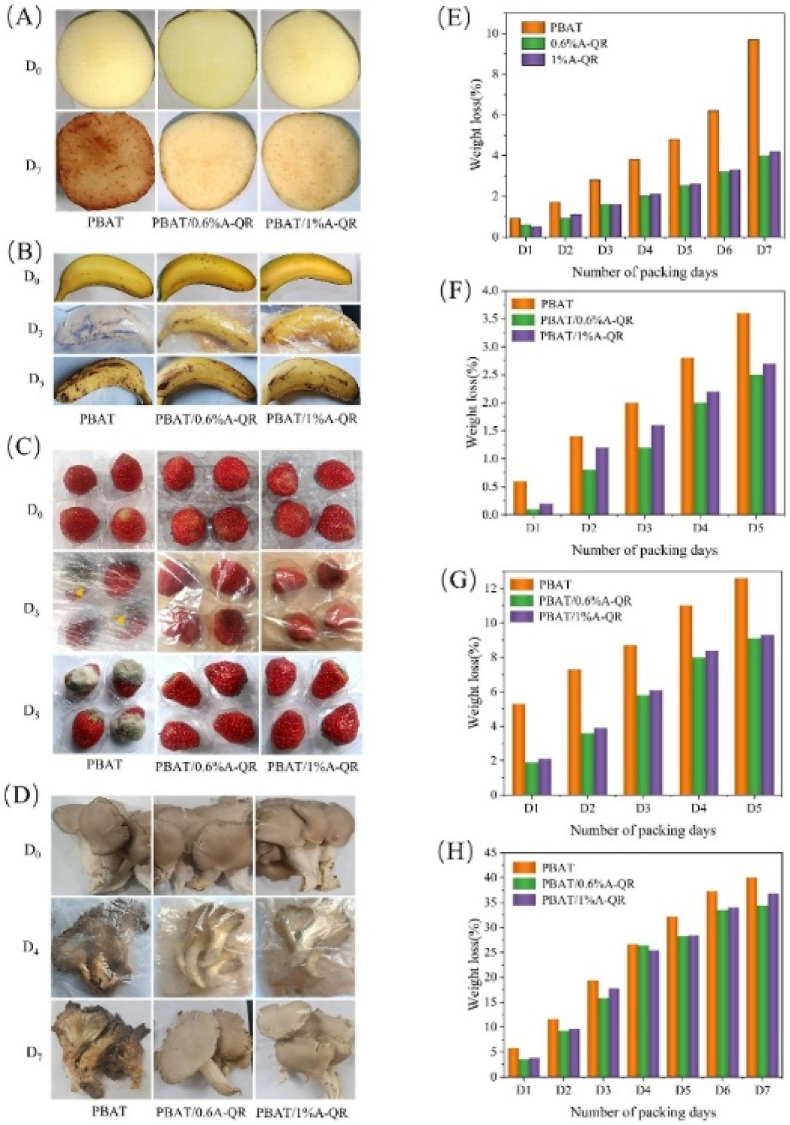
**Food storage studies: (A), (B), (C), and (D) show the storage process of strawberries, bananas, fresh-cut apples, and oyster mushrooms, respectively. (E), (F), (G), and (H) represent the weight loss rates of strawberries, bananas, fresh-cut apples, and oyster mushrooms, respectively. Reprinted with permission from ref**(Huang et al., 2023). **copywriter (2023) Elsevier.**

Huang et al. (2023) fabricated bio-based functional composite films by grafting quercetin (Qr) onto PBAT using an epoxy chain extender (ADR-4468) for active food packaging applications. The films were produced using twin-screw extrusion for compounding and single-screw film blowing, aligning with standard industrial methods, though currently restricted to pilot-level. The resulting films showed a significant enhancement in mechanical strength, with TS increasing from 19.7 MPa to 37.9 MPa (92.4 %) and EAB reaching 675.4 %. Barrier performance was improved, with WVP reduced by 215.5 % compared to PBAT/Qr composites without ADR-4468. The films exhibited strong antimicrobial activity, with inhibition zones of 42 mm for *Staphylococcus aureus* and 40 mm for *Escherichia coli* at 1 % A-Qr loading. This was attributed to the synergistic interaction between Qr and ADR-4468, strengthening the antimicrobial network. Antioxidant capacity reached 65 % DPPH radical scavenging due to Qr's polyphenolic content. Migration tests confirmed safety, with total migration ranging from 0.1 mg/dm^2^ in deionized water to 0.7 mg/dm^2^ in 10 % ethanol, well below the regulatory limit of 10 mg/dm^2^. In practical use, the films extended the shelf life of bananas, strawberries, apples, and oyster mushrooms by reducing weight loss and decay over 7 days (Fig. 3). To enable continuous large-scale manufacturing, further refinement of the processing approach is needed. With proper evaluation of production conditions and long-term stability, the films present a sustainable and versatile packaging option with significant potential for commercialization (Huang et al., 2023).

**Fig. 4:**
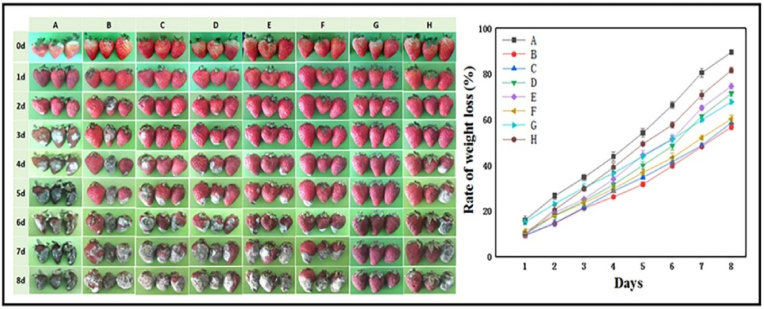
**Strawberry preservation using various films: (A) Control group, (B) Pure PVDF film, (C) 15 % ZIF-L film, (D) 15 % quercetin-loaded ZIF-L film, and (E**–**H) films containing 1 %, 5 %, 10 %, and 15 % GO@AgNPs incorporated into the 15 % Quercetin@ZIF-L film. Reprinted with permission from ref**(Yin et al., 2024). **copywriter (2024) Elsevier.**

In 2024, Yin et al. designed a nanocomposite film composed of Qr@ZIF-L/GO@AgNPs for room-temperature strawberry preservation. Solution casting was used to manufacture the films on a small scale in the lab, along with an automated coating machine for blade coating. This experimental-scale approach is suitable for early-stage research but does not represent industrial-scale manufacturing conditions. As no continuous processing methods were employed, further studies are necessary to evaluate scalability and enable large-scale production. Improvements in the film's mechanical, thermal, and barrier functionalities were observed, with a concomitant enhancement in hydrophilicity, as evidenced by a decline in the WCA from 110.4° to 57.5°. Although WVP was assessed, specific values were not reported. Robust antimicrobial performance was observed in the film, with a high Qr loading efficiency of 96 % and a pH-sensitive release profile under acidic conditions. The observed antimicrobial activity was predominantly attributed to cell membrane disruption and ROS formation. Migration studies confirmed safety, with silver ion levels in strawberries measured at 2.09 μg/L, which is well within the EU safety limit of 50 μg/L. Zinc ions were present at 1766.60 μg/L, a concentration considered beneficial to health. However, cytotoxicity and biodegradation assessments were not performed, highlighting the need for further safety evaluation. In application, the film extended strawberry shelf life to 8 days, reducing weight loss to about 70 % compared to 90 % in untreated controls (Fig. 4). The experimental data point to the Qr@ZIF-L/GO@AgNPs film as a viable, sustainable approach for fruit preservation, although further work is necessary to improve nanoparticle distribution and extend its application to a variety of food products (Yin et al., 2024).

**Fig. 5:**
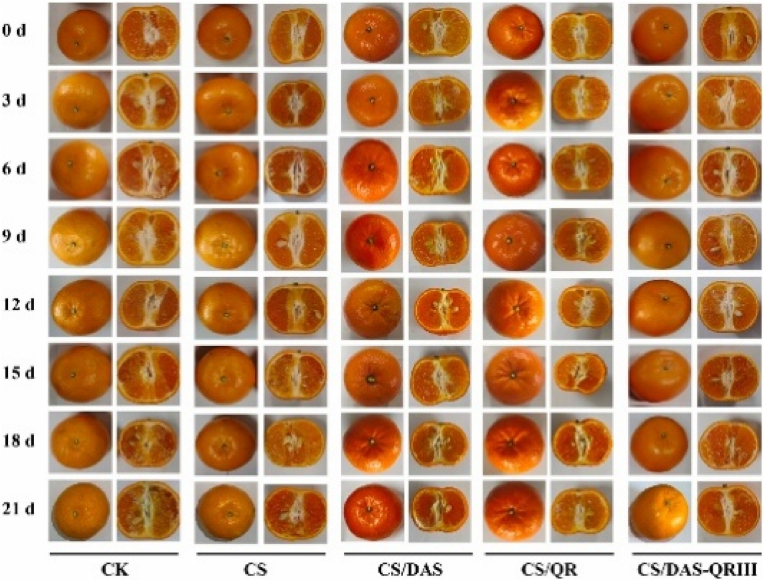
**Visual representation of films used for citrus preservation. Reprinted with permission from ref**(Sun et al., 2024). **copywriter (2024) Elsevier**.

Sun et al. synthesized multifunctional fruit packaging films by incorporating starch aldehyde-quercetin (DAS-Qr) conjugates into CS coatings in 2024. The films were fabricated at the bench scale using a solution casting technique followed by manual drying at 55 °C for 2.5 h, a stepwise process that does not fully reflect industrial-scale manufacturing. While the approach is adaptable, additional optimization is necessary for large-scale production. The developed composite films displayed considerable gains in strength, permeability resistance, and heat durability. The TS of CS/DAS-Qr III reached 18.14 MPa, compared to 10.19 MPa for pure CS, while WVP decreased to 1.67 × 10^−11^ g m^−1^·s^−1^·Pa^−1^, reflecting enhanced barrier performance. Thermal analysis showed reduced weight loss, indicating improved stability. The films showed pronounced antimicrobial activity against *Staphylococcus aureus*, with CS/DAS-Qr III reaching a 96.3 % inhibition rate, presumably due to Qr's membrane-disrupting properties. Antioxidant performance was also enhanced, with DPPH and ABTS radical scavenging activities of 80.7 % and 62.1 %, respectively, owing to Qr's polyphenolic structure. While the method is adaptable, scaling up will require further optimization. In application, CS/DAS-Qr III effectively preserved citrus fruits by limiting weight loss to 58.03 % and maintaining firmness, total soluble solids, and titratable acidity over a 21-day storage period (Fig. 5). The analysis indicates that these films are promising candidates for active packaging, with future research needed to enable industrial-scale production and broader food system applications (Sun et al., 2024).

In 2024, Zhang et al. developed a KGM/PCL) bilayer film incorporated with Qr-loaded melanin-like nanoparticles (Q@MNPs) for dual-mode synergistic bactericidal food packaging. Melt extrusion was used for the PCL layer and solution casting for the KGM layer in the research-scale fabrication of the films. Enhanced mechanical properties were observed in the film, with a TS of 30.19 MPa and an EAB measuring 42.21 %. It demonstrated improved barrier properties, with a WVP of 3.92 g mm/m^2^ day kPa and a WCA of 124.06°. Thermal stability analysis showed that the film had a degradation temperature of 317 °C. Under NIR, the film effectively reduced the survival rates of *S. aureus* and *E. coli* to 10.53 % and 12.89 %, respectively. The antioxidant activity was confirmed, with DPPH and ABTS radical scavenging rates of 70.87 % and 91.08 %, respectively. The cumulative release of Qr was 9.67 % in 95 % ethanol and 10.48 % in 10 % ethanol under NIR irradiation. The biodegradability assessment indicated that KGM and KGM-Q@MNPs films completely degraded within 7 days, while KGM/PCL and KGM-Q@MNPs/PCL films took 10 days (Fig. 6 (A)).

**Fig. 6 (A):**
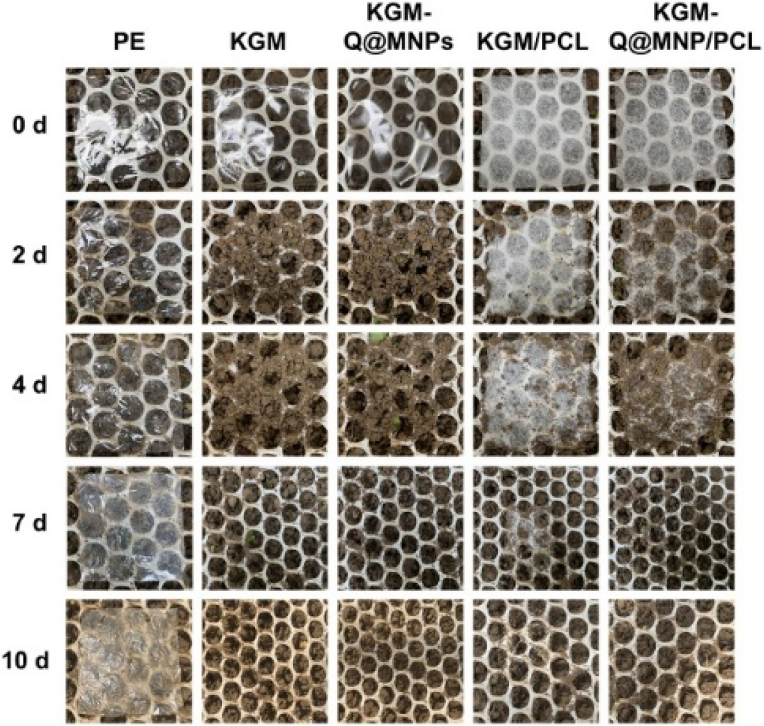
**Shows the appearance of PE, KGM, KGM/PCL, KGM-Q@MNPs, and KGM-Q@MNPs/PCL films after 10 days in natural soil. Reprinted with permission from ref**(Zhang et al., 2024). **copywriter (2024) Elsevier**.

In food packaging, the film extended cherry tomato shelf life, preserving color and texture, with hardness of 3.62 N after 14 days, weight loss at 10.54 %, and TSS change of 4.2 (Fig. 6(B)). The KGM-Q@MNPs/PCL bilayer film is a viable, sustainable packaging material, according to the study's findings. Future research will concentrate on expanding production and finding uses for additional perishable goods (Zhang et al., 2024).

**Fig. 6 (B):**
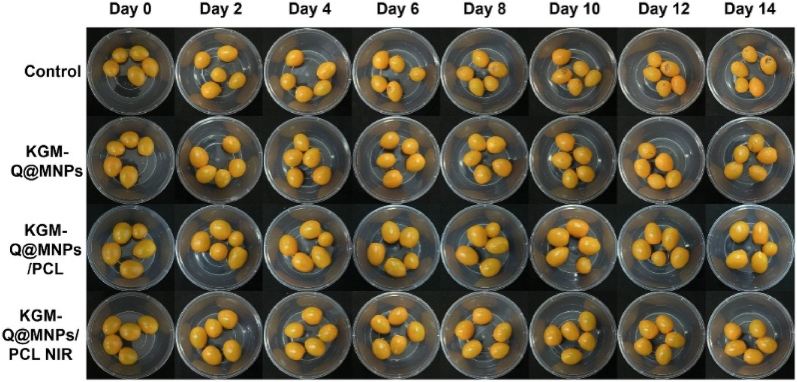
**Images of cherry tomatoes stored at 25 °C for 14 days under four distinct packaging conditions: a control film, a KGM-Q@MNPs film, a KGM-Q@MNPs/PCL film, and a KGM-Q@MNPs/PCL film enhanced with NIR. Reprinted with permission from ref**(Zhang et al., 2024). **copywriter (2024) Elsevier**.

**Fig. 7:**
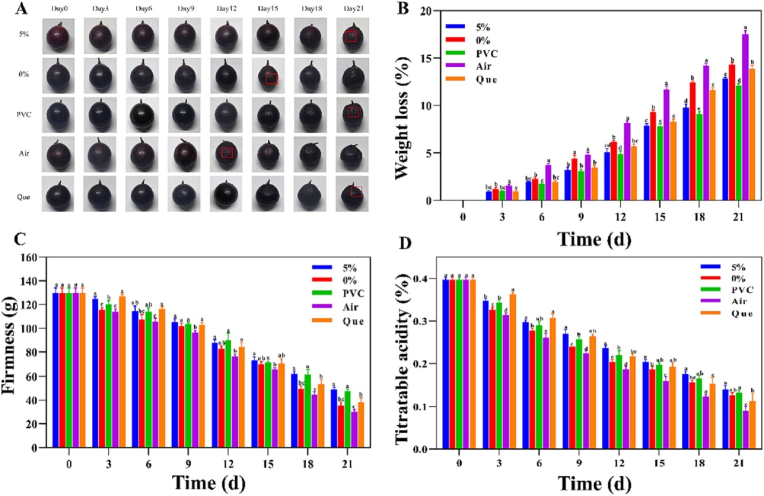
**Impact of various preservation methods on grape appearance (A), weight loss rate (B), firmness (C), and titratable acidity (D). Data are expressed as mean** ± **SD from three measurements. Different letters (a**–**e) denote significant differences among formulations at the same time point (p < 0.05). Reprinted with permission from ref**(Zeng et al., 2024). **copywriter (2024) Elsevier**.

Zeng et al. (2024) fabricated a bioactive edible film by embedding Qr-encapsulated carboxymethyl lotus root starch NPs into a lotus root starch matrix using solution casting, a micro-scale approach typical of preliminary research. Although the method is potentially adaptable for industrial use, further development is required for continuous, high-throughput production. The incorporation of 5 % QNPs resulted in enhanced mechanical, barrier, and thermal properties. The film exhibited a 23 % increase in TS, reaching 5.32 MPa, and a WVP of 1.47 × 10^−8^ g m/m^2^·s·kPa, indicating improved moisture resistance. Thermal stability was also enhanced, with a maximum degradation temperature of 305.77 °C. The film demonstrated effective antifungal activity against *Botrytis cinerea*, showing inhibition zones of 11.93 ± 0.32 mm. This effect was linked to Qr's ability to disrupt cell membranes and inhibit vital fungal processes. Antioxidant capacity was notably enhanced, with DPPH and ABTS radical scavenging activities reaching 68.68 % and 74.41 %, respectively. Applied to grape preservation, the film reduced weight loss to 12.8 % over 21 days, compared to 17.5 % in untreated samples, and maintained fruit firmness, effectively extending shelf life (Fig. 7). The research concludes that QNPs and starch films are environmentally benign and promising fruit preservation packaging materials, deserving of more investigation into their scalability and wider range of food uses (Zeng et al., 2024).

**Fig. 8:**
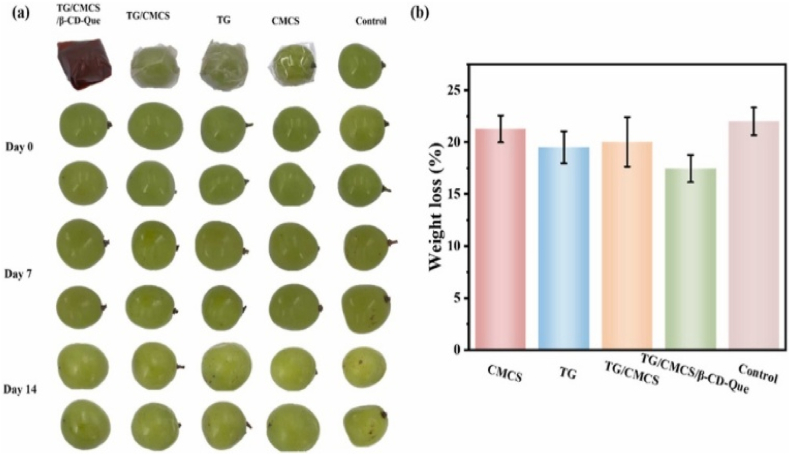
**Images of grapes packaged with different films at the start and after 14 days of storage; (b) Weight loss rate of green grapes after 14 days of storage. Reprinted with permission from ref**(Liu et al., 2025). **copywriter (2025) Elsevier**.

In 2025, Liu et al. designed a food packaging film by encapsulating quercetin within β-CD to form inclusion complexes, which were then dispersed into a TG and CMCS matrix using the solution casting method at the research scale. This modular, batch-based approach is typical in experimental research but unsuitable for industrial production, which would require adaptation to continuous methods such as roll-to-roll coating or extrusion. The TG/CMCS/β-CD-Qr films showed improved mechanical properties, with TS increasing from 14.36 MPa to 15.03 MPa and EAB from 13.68 % to 25.74 %, attributed to hydrogen bonding between β-CD-Qr and the polymer matrix. Barrier performance was enhanced, with WVP decreasing from 3.50 to 3.18 g mm/m^2^·h·kPa and oxygen permeability reducing from 0.40 to 0.35 × 10^−4^ g/m^2^·day·kPa due to a denser film structure. Thermal stability improved as β-CD-Qr reinforced intermolecular interactions, slowing weight loss in thermogravimetric analysis. Exhibiting remarkable antimicrobial potency, the film effectively inhibited *E. coli* and *S. aureus* by 99.40 % and 99.23 %, respectively. Complementing its antibacterial capabilities, the film also showcased strong antioxidant activity, with radical scavenging rates of 78.01 % DPPH and 99.59 % ABTS, highlighting its multifunctional properties. For maintaining the freshness of green grapes, the film reduced weight loss to 17.46 % over 14 days (Fig. 8). TG/CMCS/β-CD-Qr films demonstrate potential as sustainable and versatile packaging solutions, though further investigation is necessary to facilitate large-scale industrial implementation (Liu et al., 2025).

**Fig. 9:**
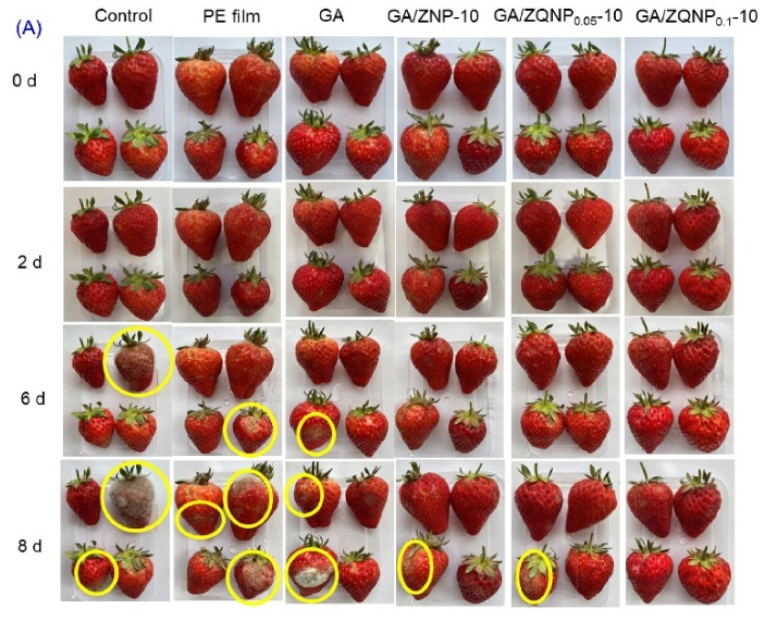
**Change in strawberry appearance from day 0 to day 8 with different films: control, PE film, GA, GA/ZNP-10, GA/ZQNP_0.05_-10, and GA/ZQNP_0.1_-10. Reprinted with permission from ref**(Xu et al., 2025). **copywriter (2025) Elsevier**.

Xu et al. (2025) engineered gelatin films incorporated with zein-quercetin nanoparticles (GA/ZQNPs) to enhance mechanical, barrier, and thermal properties for food packaging applications using solution casting followed by air drying at the small-scale for 48 h via a manual process typical of laboratory studies, without industrial or continuous equipment. While this method shows potential for scale-up, further research is required to develop efficient large-scale manufacturing techniques. The GA/ZQNP_0.1_-10 films showed a TS of 3.2 MPa, 1.39 times higher than pure gelatin, and an EAB of 142 %. WVP decreased by 78.4 % (1.9 ± 0.3 g mm/(m^2^·h·kPa) and oxygen permeability by 76.9 % (12.76 × 10^−4^ mol/m^2^·s), indicating enhanced barrier performance. Thermal stability improved, with 14.4 % residue remaining at 800 °C. Strong antimicrobial effects were observed in the films, with inhibition zones of 45.3 ± 1.2 mm for *E. coli* and 48.7 ± 1.1 mm for *Penicillium* sp., which can be ascribed to the membrane-disrupting action of Qr. Antioxidant activity was confirmed by DPPH scavenging (64.9 ± 0.7 %) and FRAP assays. Application tests showed effective strawberry preservation, extending shelf life to 8 days with minimal decreases in hardness (22.4 ± 1.7 %), weight loss (12.5 ± 0.7 %), and total soluble solids (8.5 ± 0.5 %) (Fig. 9). These results indicate that GA/ZQNP films are promising sustainable packaging materials, though future work should focus on optimizing migration, cytotoxicity, and biodegradability for wider use (Xu et al., 2025).

### 5.2 Packaging for meat and seafood products

In 2020, Khan et al. synthesized edible gelatin composite films enriched with polyphenol-loaded NEs for chicken meat packaging using solution casting at the laboratory scale. Although effective for a feasibility study, this manual process lacks the throughput and consistency needed for industrial production. However, with further optimization, the method shows potential for scale-up. Mechanical properties of the films were significantly enhanced, with TS increasing from 9.81 ± 0.20 MPa (control) to 34.82 ± 0.39 MPa upon incorporation of 20 % curcumin NE. Correspondingly, EAB improved from 41.02 ± 1.40 % to 73.01 ± 0.93 %, while the EM rose from 103.02 ± 1.90 MPa to 146.76 ± 0.96 MPa. Barrier properties improved, with WS decreasing from 61.09 ± 0.02 % (control) to 46.72 ± 0.02 % (20 % curcumin NE), whereas WVP increased from 12.37 × 10^−10^ g s^−1^ m^−1^ Pa^−1^ to 21.58 × 10^−10^ g s^−1^ m^−1^ Pa^−1^, indicating higher moisture retention. The films demonstrated significant antimicrobial activity, with inhibition zones of 7.47 ± 0.09 mm against *E. coli* and 6.97 ± 0.03 mm against *Salmonella typhimurium* for 20 % curcumin NE, attributed to the interaction of phenolic compounds with bacterial cellular structures, inhibiting microbial growth. Antioxidant activity, measured via DPPH inhibition, reached 60.51 ± 0.36 % for 20 % curcumin NE, compared to 1.07 ± 0.01 % for control films. The films were applied to fresh broiler meat, extending shelf life from 10 days (control) to 17 days (20 % curcumin NE), with microbial counts remaining below 10^6^ CFU/g, meeting food safety standards. This study highlights the promise of polyphenol-loaded gelatin films as biodegradable, antioxidant, and antimicrobial packaging alternatives. However, further investigation into formulation refinement, long-term stability, safety validation, and commercial-scale production techniques is essential to realize their full industrial potential (Khan et al., 2020).

**Fig. 10:**
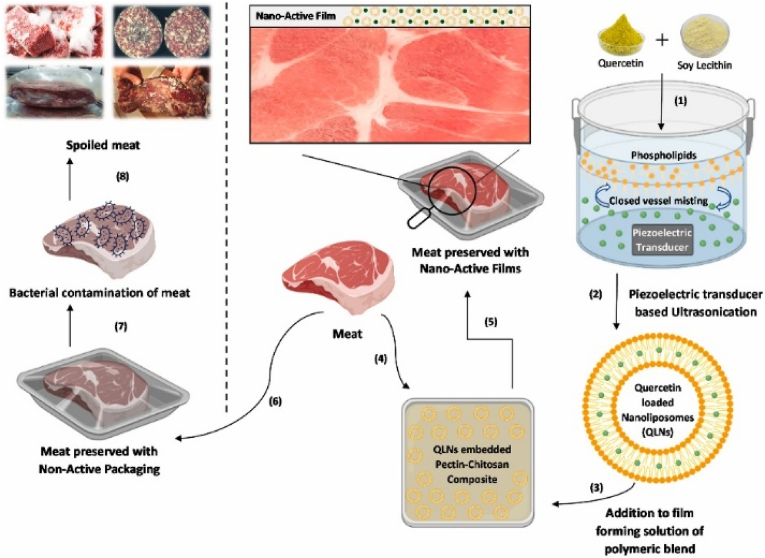
**Schematic diagram illustrating quercetin-loaded nanoliposomes integrated into nano-active packaging films for efficient food preservation against bacterial spoilage. Reprinted with permission from ref**(Afroz Ali et al., 2023). **copywriter (202**3**) Elsevier**.

Afroz Ali et al. engineered pectin-CS-based nanocomposite films embedded with QNLs to enhance food packaging properties (Fig. 10) in 2023. The films were prepared at lab scale via solution casting and air-dried at 25 °C for 48–72 h. While appropriate for research, this batch-wise method lacks industrial throughput but is adaptable for potential scale-up. The nano-active films showed significantly improved mechanical properties, with TS increasing from 2.49 ± 0.02 MPa to 9.74 ± 0.26 MPa, and YM rising from 5.04 ± 0.04 MPa to 24.17 ± 0.64 MPa. Barrier performance was also enhanced, as WVP decreased from 6.54 ± 0.42 × 10^−11^ g m^−1^·s^−1^·Pa^−1^ in non-active films to 3.73 ± 0.31 × 10^−11^ g m^−1^·s^−1^·Pa^−1^ in nano-active films. Thermal analysis (TGA and DSC) indicated weight loss at 125–250 °C due to liposomal degradation and 250–500 °C due to polymer decomposition. The films effectively reduced bacterial populations, achieving a 2-log reduction in *Escherichia coli* and a 1.7-log reduction in *Listeria monocytogenes* over 96 h, attributed to the sustained release of quercetin alongside CS's native antimicrobial activity. Application of the films to chicken fillets resulted in a marked reduction in microbial counts over a 4-day refrigeration period, effectively extending shelf life. These observations show the potential of QNL-incorporated pectin-CS films as a biodegradable solution for active food packaging, though further studies are needed to enhance their scalability and commercial feasibility (Afroz Ali et al., 2023).

In 2024, Bakeshlou et al. fabricated wheat gluten nanocomposite films with Qr nanoliposomes (10 % v/v) and ZnO NPs (6 % w/w) for Rainbow trout packaging. Prepared via lab-scale homogenization, casting, and drying, the method suits research but needs further development for industrial scalability. The study assessed the film's physicochemical properties and its impact on fish preservation over 6 days. The nanocomposite film exhibited enhanced antioxidant and antimicrobial activity, significantly reducing the peroxide index, TBARs, and total TVB-N levels in the packaged fish. Its antimicrobial efficacy was demonstrated by a substantial reduction in total microbial counts compared to the control, attributed to the synergistic action of Qr nanoliposomes and ZnO NPs in disrupting microbial membranes and inhibiting enzymatic activity. The film also effectively delayed lipid oxidation, as evidenced by the lower peroxide and TBARs values, thereby helping preserve the freshness of the fish. Color analysis revealed that the packaged fish appeared slightly duller than the control, while no significant difference in fat content was observed between the treated and untreated samples. Overall, incorporating ZnO NPs and Qr nanoliposomes enhanced the film's protective properties, making it a promising candidate for extending the shelf life of perishable foods such as Rainbow trout. Future research should focus on scaling up production, optimizing nanoparticle dispersion, and exploring applications in packaging other perishable food products (Bakeshlou et al., 2024b).

Mirzaei et al. (2024) developed pH-sensitive hydrogel films using CG incorporated with quercetin (QUE) or ELE for monitoring chicken meat freshness. κ-Carrageenan hydrogel films were lab-prepared via manual solution casting with QUE or ELE, followed by drying at 40 °C for 24 h. Due to stronger intermolecular interactions in CG-ELE compared to CG-QUE, the films exhibited enhanced mechanical properties, with CG-ELE displaying higher TS (13.2 ± 0.6 MPa) and reduced EAB (5 ± 0.1 %). Barrier properties were improved, with transparency levels above 90 % for films containing 0.3 % QUE or ELE, ensuring visibility and consumer confidence. Thermal stability was maintained, with no significant degradation observed up to 200 °C, suitable for food packaging applications. Antimicrobial activity was significant, with minimum inhibitory concentration (MIC) values of 8–128 μg/mL for CG-QUE and 4–128 μg/mL for CG-ELE against spoilage bacteria like *S. aureus* and *E. coli*, due to QUE's ability to disrupt microbial cell membranes and ELE's additional bioactive compounds. When applied to packaging chicken meat, the films effectively monitored spoilage by measuring total volatile basic nitrogen (TVB-N) levels. After 3 days, CG-QUE and CG-ELE films recorded TVB-N values of 29.75 ± 0.02 mg/100g and 26.5 ± 0.04 mg/100g, respectively, which were lower than those in control samples. This indicates delayed spoilage. By exhibiting strong performance in freshness monitoring and spoilage inhibition, CG-ELE films hold significant potential as an environmentally conscious alternative to traditional plastic-based packaging. Future investigations should focus on standardizing extract composition, improving mechanical properties, and refining fabrication techniques to facilitate large-scale production (Mirzaei et al., 2024).

### 5.3 Packaging of oil and high-fat food

In 2019, Braga et al. synthesized PVC-based films with 0.4 % Qr and AgNPs using a lab-scale solvent casting method. The incorporation of AgNPs markedly influenced the films' mechanical behavior. TS climbed from 2.6 ± 0.2 MPa in PVC-Qr to a peak of 9.3 ± 0.4 MPa at 1 % AgNP, before modestly declining to 7.9 ± 0.1 MPa at 8 % loading. Meanwhile, EAB showed a steep decrease with increasing AgNP concentration, falling from 203 ± 22 % to just 19 ± 9 %. Notably, the 1 % AgNP films retained a substantially higher EAB of 94 ± 36 %, suggesting a more balanced compromise between strength and flexibility at this concentration. The films’ UV-light barrier properties improved significantly, with UV transmission decreasing from 53.77 ± 11.45 % (PVC) to 1.07 ± 0.23 % (PVC-Qr + AgNP 8 %) at 250 nm, making them highly effective in blocking harmful radiation. Thermal stability, assessed via TGA, showed a two-step degradation process, with the major degradation occurring between 276 °C and 363 °C, and AgNP incorporation slightly shifted the degradation temperature to higher values, indicating improved thermal resistance. Antimicrobial activity against *Listeria monocytogenes*, *E. coli*, and *Salmonella typhimurium* was highly effective, with complete inhibition observed in films containing AgNPs, attributed to the release of Ag^+^ ions disrupting bacterial cell membranes. Antioxidant activity, measured via DPPH radical scavenging, reached 60.44 ± 6.36 % for PVC-Qr films after 24 h but decreased to 29.45 ± 8.66 % with 8 % AgNPs, likely due to Qr-AgNP complexation, reducing available hydroxyl groups for radical scavenging. These films show potential for food packaging applications, particularly for fatty foods, by reducing lipid oxidation and microbial contamination, while also providing promising UV barrier properties and mechanical strength. Future research should optimize AgNP concentrations for better antioxidant activity and explore real-food shelf-life studies to validate practical applications (Braga et al., 2019).

Tongdeesoontorn et al. (2020) designed cassava starch-CMC films with Qr and TBHQ via solution casting, which is suitable for lab-scale but limits industrial scalability. The mechanical performance of the films improved, as TS rose from 12.5 MPa (control) to 14.2 MPa with the addition of 50 mg Qr. At 34 % relative humidity (RH), EAB declined from 45 % to 35 %. However, at 54 % RH, EAB increased to 55 % in the TBHQ-containing film, suggesting a water-induced plasticizing effect. WS decreased from 78 % (control) to approximately 60 % with the incorporation of Qr and TBHQ, enhancing barrier properties. Thermal analysis revealed a decrease in melting temperature (T_m_) from 128.65 °C (control) to 110.09 °C for TBHQ-containing films, while the heat of fusion (ΔH) increased from 154.14 J/g (control) to 227.88 J/g for films with 200 mg Qr, indicating modified crystallinity. The films demonstrated strong antioxidant activity, with total phenolic content increasing to 0.8 mg GAE/g (Qr), while DPPH scavenging activity remained stable over 30 days. In food applications, the films effectively extended the shelf life of lard by reducing peroxide values from 20 meq/kg (control) to 10 meq/kg (Qr-containing films) over 70 days, and maintained pork redness above 80 % after 12 days, confirming their efficacy in delaying oxidation. Although scalable techniques like extrusion or roll-to-roll coating exist, they were not used here. Further research is needed to adapt the formulation and optimize processing conditions for industrial-scale manufacturing. While the results are promising, successful commercialization will require additional scale-up studies that also address broader food applications, migration properties, and long-term, eco-friendly performance (Tongdeesoontorn et al., 2020).

In 2023, Jakubowska et al. developed active packaging films based on chitosan (Ch), plasticized with a DES composed of choline chloride and citric acid, and incorporated with quercetin (QUE) for rapeseed oil storage. Produced by lab-scale solution casting and dried at 30 °C for 48 h, this method is not scalable for industry. While techniques such as extrusion and roll-to-roll coating are available for large-scale production, they were not employed in this study. The films exhibited enhanced mechanical properties, with TS increasing from 18.7 MPa (Ch/DES) to 23.0 ± 1.4 MPa (Ch/DES/QUE3) and YM increasing by 35 % with 3 wt% QUE, though EB decreased due to QUE's hydrophobic nature. Barrier properties improved, with the WVTR decreasing from 16.2 g m^−2^·h^−1^ (Ch/DES) to 12.8 g m^−2^·h^−1^ (Ch/DES/QUE3), and the oxygen transmission rate (OTR) also reduced, enhancing oxidative stability. Thermal stability remained consistent, with QUE addition not significantly affecting degradation temperatures. The antimicrobial activity of the films was ascribed to the intrinsic properties of Ch and the membrane-disrupting action of QUE, although precise bactericidal rates were not determined. Antioxidant activity significantly increased, with DPPH radical scavenging activity rising from 48.0 % (Ch/DES) to 71.8 % (Ch/DES/QUE3) and hydroxyl radical scavenging from 51.1 % to 81.8 %, due to QUE's phenolic hydroxyl groups. In food packaging applications, the films extended the shelf life of rapeseed oil, reducing peroxide value (PV) by 67 % and anisidine value (AnV) by 50 % after 4 weeks of storage, maintaining the TOTOX index below 10. The study concludes that Ch/DES/QUE films are promising for active food packaging, particularly for oils, fruits, and vegetables, with future research needed to optimize QUE loading and explore large-scale production feasibility (Jakubowska et al., 2023).

### 5.4 Packaging for bakery and starchy foods

In 2019, Bai et al. prepared antioxidant and Al^3+^-sensing films by incorporating Qr into carboxymethyl CS via lab-scale solution casting. The mixture was poured into Plexiglas plates, dried at 28 °C for 2 days, and conditioned at 50 % humidity. This manual batch process lacks industrial scalability. The incorporation of 2.5 and 5 wt% Qr did not significantly change the TS, while 7.5 wt% Qr reduced it due to the formation of Qr crystals. CMCS-Qr II (5 wt% Qr) showed the highest TS (29.14 MPa) compared to CMCS film (26.26 MPa). While the addition of Qr did not significantly alter the WVP of CMCS films, CMCS-Qr II demonstrated the lowest value (15.16 × 10^−11^ g m^−1^ s^−1^ Pa^−1^). Thermal stability increased, with CMCS-Qr films showing higher maximum weight loss temperatures (283–291 °C) than CMCS films (267 °C). Marked antioxidant performance was observed in the films, releasing 17.67–19.44 mg/g flavonoids in aqueous and 2.26–8.98 mg/g in fatty food simulants. Notably, CMCS-Qr III (7.5 wt%) achieved 100 % DPPH radical scavenging in water, while efficacy diminished in ethanol. The films also demonstrated Al^3+^-sensing ability, with UV–Vis absorption peaks shifting from 254 to 367 nm to 266 and 430 nm upon Al^3+^ interaction, indicating the potential for detecting Al^3+^ in food. Migration tests indicated sustained release of Qr. The films showed promise for food packaging applications, particularly for foods prone to oxidation and Al^3+^ contamination, such as deep-fried dough sticks and steamed buns. Although methods like extrusion or roll-to-roll coating could be explored for industrial adaptation, further research is needed to assess scalability and commercial feasibility. Future studies should validate the films’ performance in real food systems and explore their potential as biodegradable and intelligent packaging materials (Bai et al., 2019).

### 5.5 Intelligent packaging for food freshness monitoring

In 2018, Marrez et al. fabricated eco-friendly cellulose acetate nanocomposite films embedded with green-synthesized AgNPs using polyphenolic compounds for antibacterial food packaging. Solution casting was employed to produce these films on a lab scale, drying them at room temperature in petri dishes or glass plates via a manual batch process without industrial-scale equipment. Elevated barrier properties were confirmed by low swelling ratios (0.28–0.65) and moderate water content (25–36 %), indicating limited hydrophilicity suitable for food packaging. The films effectively inhibited the growth of various foodborne pathogens, producing inhibition zones of 25.5 mm (*Staphylococcus aureus*), 18.2 mm (*Bacillus cereus*), 21.2 mm (*Escherichia coli* and *Salmonella typhi*), 11.2 mm (*Pseudomonas aeruginosa* and *Pseudomonas fluorescens*), and 20.5 mm (*Klebsiella pneumoniae*). The MIC values of AgNPs ranged between 16.7 and 46.7 μg/mL, confirming their potent antibacterial efficacy. The antimicrobial mechanism involved the release of Ag^+^ ions, which disrupt bacterial cell membranes and inhibit enzymatic activity. The films also exhibited antioxidant activity, though specific values were not provided. Silver migration tests revealed that the released AgNPs were below the permissible limit of 10 ppm, with values as low as 0.027 ppm for Qr-based films. Cytotoxicity tests using brine shrimp showed low toxicity, with survival rates above 82.5 % after 72 h. Overall, the study highlighted the strong potential of CA-AgNP nanocomposite films for active food packaging, owing to their excellent antibacterial properties, low toxicity, and compliance with migration standards. Future research should focus on evaluating their thermal and mechanical properties, biodegradability, and effectiveness in real food packaging applications, including shelf-life extension for different food products. Furthermore, refining the formulation for wider food uses and assessing long-term safety and environmental impact are vital for successful commercial development (Marrez et al., 2018).

Roy and Rhim (2021) developed CS-based bioactive nanocomposite films reinforced with Qr-loaded CS NPs for active food packaging. The films were fabricated in the laboratory using solution casting on Teflon-coated glass plates, then dried at room temperature for 48 h and conditioned at 25 °C with 50 % humidity. This pilot-scale, manual process is not adaptable to industrial production. The mechanical properties of the films were enhanced, as TS increased from 62.2 ± 6.3 MPa for pure CS to 72.7 ± 5.0 MPa for Chs/QCNP1, while EAB stayed consistent at around 5.9 ± 1.4 %. Barrier properties showed a slight increase in WVP from 3.95 × 10^−10^ g m/m^2^·Pa·s (neat CS) to 4.71 × 10^−10^ g m/m^2^·Pa·s (Chs/QCNP3), and thermal stability improved, with maximum decomposition temperature (T_max_) increasing from 270 °C to 280 °C. Moderate antimicrobial activity was observed in the films, effectively reducing *E. coli* and *L. monocytogenes* viability through Qr's disruption of bacterial membranes and CS's polycationic properties. Due to Qr's polyphenolic compounds, antioxidant activity significantly increased, with DPPH and ABTS radical scavenging reaching 93.2 ± 2.0 % and 80.0 ± 1.5 %, respectively. Migration tests revealed Qr release was highest in 3 % acetic acid (12 μg/mm^2^) and 50 % ethanol (10 μg/mm^2^), indicating suitability for acidic and alcoholic food simulants. The films showed potential for extending the shelf life of UV-sensitive foods, with UV-blocking properties reducing transmittance at 280 nm (T_280_) to 0.9 ± 0.3 %. The CS/QCNP composite films present a viable biodegradable alternative for active packaging, exhibiting notable improvements in mechanical, antimicrobial, and antioxidant performance. However, additional optimization is needed before they can be effectively applied in real-world settings (Roy and Rhim, 2021).

**Fig. 11:**
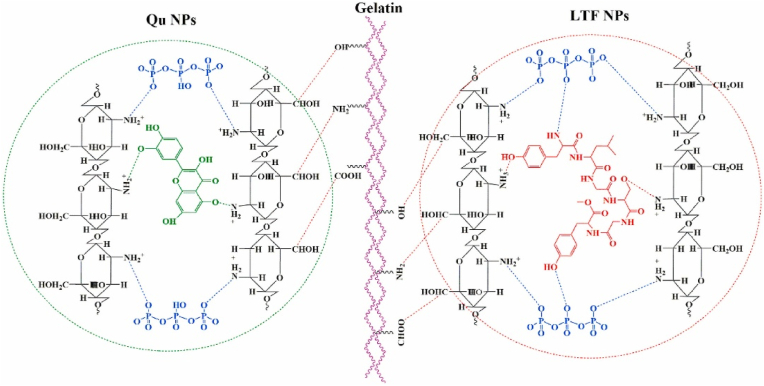
**The proposed hypothesis on the molecular interactions among gelatin, Qu NPs, and LTF NPs. Reprinted with permission from ref** (Tavassoli et al., 2021). **copywriter(2021) Elsevier**.

In 2021, Tavassoli et al. designed multifunctional gelatin-based nanocomposite films containing Qr, lactoferrin, and CS nanofibers via solution casting for active food packaging (Fig. 11). Though promising, the lab-scale method limits industrial scalability. Scalable techniques like extrusion need to be explored, along with improvements in interlayer adhesion. The mechanical properties were altered, with TS decreasing from 65.1 ± 7.6 MPa (pure gelatin) to 19.4 ± 0.7 MPa (G/Qu/LTF), and EAB increasing from 2.19 ± 0.4 % to 29.9 ± 1.7 %, indicating reduced stiffness but enhanced flexibility due to nanoparticle incorporation. Barrier properties improved, with WVP decreasing from 8.25 × 10^−12^ g m/m^2^·s·Pa (pure gelatin) to 7.50 × 10^−12^ g m/m^2^·s·Pa (G/Qu), suggesting improved moisture resistance. Thermal analysis confirmed structural stability up to 200 °C, with degradation peaks occurring around 242 °C, 401 °C, and 582 °C, attributed to the thermal resistance of Qr and lactoferrin. The films exhibited strong antimicrobial activity, with inhibition zones of 17.8 ± 1.6 mm (*E. coli*) and 20.5 ± 0.5 mm (*S. aureus*), attributed to Qr-induced membrane disruption and lactoferrin's iron-chelating effect. Antioxidant properties were significantly enhanced, with DPPH scavenging reaching 93.2 ± 2.0 %, demonstrating Qr's high free radical scavenging efficiency. Migration tests revealed Qr release was highest in 50 % ethanol (0.25 mg/L), indicating its suitability for fatty food applications. Biodegradation analysis demonstrated complete degradation within 25 days under composting conditions, indicating excellent environmental compatibility. The films responded to ammonia vapor with noticeable color shifts, demonstrating their suitability for application as freshness indicators. However, several research gaps and future directions remain to be addressed. These include scaling up the production process for industrial feasibility, testing the films under real food packaging conditions, and improving functionality through the incorporation of additional bioactives or intelligent indicators. Exploring alternative biopolymer matrices could further enhance film properties while preserving biodegradability. Comprehensive assessments on safety, compound migration, and environmental impact are essential for regulatory compliance. Additionally, consumer acceptance, especially related to the film's color changes due to Qr, should be studied to ensure market viability (Tavassoli et al., 2021).

**Fig. 12:**
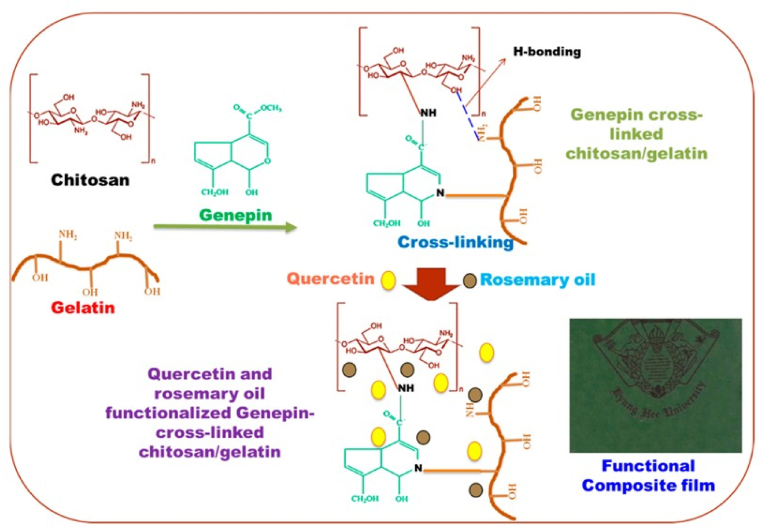
**Schematic representation of the fabrication process of biocomposite films and the interactions between polymers and fillers. Reprinted with permission from ref**(Roy and Rhim, 2022). **copywriter (2022) Elsevier**.

Roy and Rhim et al. formulated genipin-crosslinked CS/gelatin-based functional films incorporated with RO and quercetin (QCT) for active food packaging (Fig. 12) in 2022. On a small laboratory scale, the genipin-crosslinked CS/gelatin films were made by solution casting. Strengthened mechanical properties were observed in the films, with TS increasing from 77.3 MPa (neat film) to 86.7 MPa (CTS/GTN[Gen]/QCT/RO), while EAB decreased significantly from 6.3 % to 5.7 %. The WVP remained relatively unchanged (0.65-0.71 × 10^−9^ g m/m^2^·s·Pa), and the WCA indicated a slightly increased surface hydrophilicity (60.5°–61.5°). Thermal stability improved with crosslinking, as the maximum degradation temperature increased by 10 °C, while the addition of bioactive fillers had no significant impact on thermal properties. The observation revealed strong antimicrobial activity in the films, with *E. coli* and *L. monocytogenes* populations decreasing by 7.3–7.6 and 6.8–7.7 log CFU/mL, respectively, due to the combined membrane-disrupting effects of QCT and RO. Antioxidant activity significantly increased, with radical scavenging rates enhanced by QCT and RO, particularly in ABTS assays. However, crosslinking with genipin did not significantly alter antioxidant activity. The films were suitable for packaging perishable foods, extending shelf life by inhibiting microbial growth and oxidative spoilage. Additional research is required to evaluate the scalability and economic viability of film production. Overall, these multifunctional films hold potential for active food packaging, but further studies are necessary to enhance their flexibility and water vapor barrier properties to meet the standards of commercial plastic films (Roy and Rhim, 2022).

**Fig. 13:**
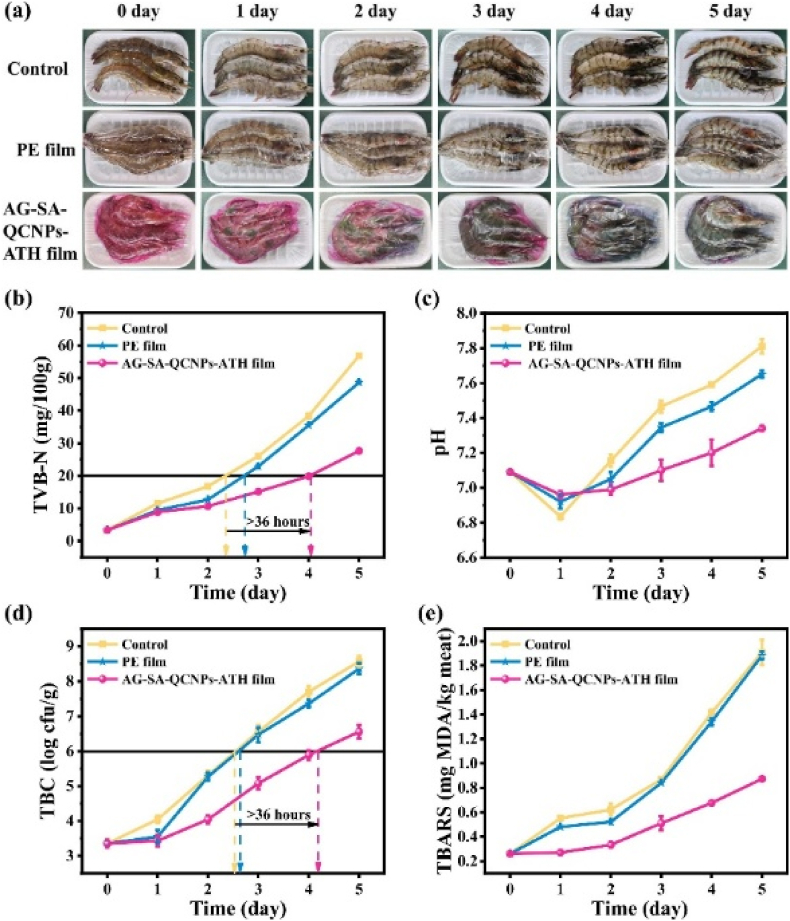
**(a) Photographic documentation of shrimp preservation: the first row shows the control group, the second row represents the PE film group, and the third row corresponds to the AG-SA-QCNPs-ATH film group. Changes in TVB-N (b), pH (c), total bacterial counts (d), and TBARS (e) are presented for shrimp that were either unpackaged, packaged with PE film, or packaged with AG-SA-QCNPs-ATH film during chilled storage. Reprinted with permission from ref** (Dong et al., 2023). **copywriter (2023) Elsevier**.

In 2023, Dong et al. synthesized a multifunctional intelligent film by integrating purple sweet potato anthocyanin (ATH) and quercetin-loaded CS nanoparticles (QCNPs) into an agar/sodium alginate (AG/SA) matrix for monitoring and preserving shrimp freshness. The AG-SA-QCNPs-ATH films were fabricated on a laboratory scale through the solution casting technique, a manual, intermittent process typically used in research settings without the involvement of industrial-scale. The film exhibited enhanced mechanical properties, with TS increasing from 8.0 MPa (AG-SA) to 12.1 MPa (AG-SA-QCNPs-ATH) and EAB reaching 278.2 %. Barrier properties improved, with WVP reduced by 47.2 % and UV transmittance significantly lowered due to QCNPs and ATH. Thermal stability was maintained, with a residual mass of 22.8 % at 600 °C. The film demonstrated strong antimicrobial activity, with bactericidal rates of 86.72 % against *Staphylococcus aureus* and 33.15 % against *Escherichia coli*, attributed to QCNPs disrupting bacterial cell walls and ATH inhibiting intracellular proteins. Antioxidant activity reached 82.66 % DPPH radical scavenging, driven by the synergistic effects of QCNPs and ATH. In food packaging applications, the film extended shrimp shelf life by 36 h at 4 °C, maintaining freshness as indicated by total volatile base nitrogen (TVB-N) levels below 20 mg/100 g for up to 3 days (Fig. 13). The film's pH-sensitive color change, quantified by ΔG/R values via smartphone RGB analysis, accurately monitored shrimp freshness. The study concludes that the AG-SA-QCNPs-ATH film offers a sustainable, multifunctional solution for intelligent food packaging, though further research is needed to improve its stability under varying temperatures and optimize production for industrial feasibility (Dong et al., 2023).

### 5.6 Packaging for low and intermolecular moisture food

In 2023, Sani et al. designed a multifunctional, biodegradable, and multifunctional packaging film composed of MC and CS NFs, incorporating ZnO NPs, quercetin (Qu), and NAT, utilizing the casting technique. The fabrication of MC/CNFs/ZNPs/Qu/NAT films was carried out at a laboratory level through a solution casting approach. This process, conducted manually in discrete batches, reflects standard research-scale practices without the use of industrial-grade equipment. TS increased from 40.4 MPa in neat MC to 66.65 MPa in MC/CNFs/ZNPs/Qu/NAT, while EAB significantly decreased from 38.25 % to 9.8 %, indicating enhanced mechanical properties of the film. Barrier properties improved significantly, with WVP reducing from 6 × 10^−10^ g m/m^2^·s·Pa (MC) to 1.85 × 10^−10^ g m/m^2^·s·Pa (MC/CNFs/ZNPs/Qu/NAT), and WCA increasing significantly from 44.6° to 97.6°, indicating enhanced hydrophobicity. Thermal stability was not significantly affected, with the film showing degradation peaks at 300 °C, mainly due to polymer decomposition. Antimicrobial activity was strong in the film, with inhibition zones measuring 18.6 mm for *E. coli*, 19.4 mm for *S. aureus*, 13.7 mm for *Aspergillus* sp., and 16.0 mm for *Penicillium* sp., attributed to ZNPs disrupting bacterial membranes and NAT targeting fungal ergosterol. Antioxidant activity reached 84.15% DPPH scavenging, primarily due to Qu's hydroxyl groups. Qu release was highest in 50 % ethanol, followed by 10 % ethanol, 90 % ethanol, and water, indicating controlled release kinetics. Biodegradability tests showed 93.5 % weight loss after 30 days in soil, indicating environmental friendliness. The film was suitable for packaging perishable foods, effectively extending shelf life by inhibiting microbial growth and oxidative spoilage. Its excellent water vapor barrier and high surface hydrophobicity make it particularly effective for low and intermediate-moisture foods by preventing moisture migration. Furthermore, its strong antimicrobial and antioxidant properties enhance preservation in moisture-sensitive environments. In conclusion, this multifunctional film offers a sustainable alternative to synthetic plastics, with future research needed to optimize its mechanical flexibility and long-term stability for various food packaging scenarios (Sani et al., 2023)

Bakeshlou et al. developed wheat gluten-based nanocomposite films incorporating Qr-loaded nanoliposomes (NL) and ZnO NPs to enhance food packaging performance in 2024. Manual fabrication of the films was carried out at the laboratory scale through mixing the components, adjusting the pH, homogenizing the solution, casting into molds, and drying under controlled conditions, all without the use of industrial equipment. At optimal concentrations of 10 % NL and 6 % ZnO NPs, the film exhibited increased mechanical properties, with TS rising to 5.90 MPa and EAB reaching 2.74 %. The thermal stability was analyzed using DSC, revealing melting temperatures of 260 °C, 240 °C, and 230 °C, and thermal degradation temperatures at 280 °C, 290 °C, and 300 °C. Barrier properties were enhanced, as WVP decreased to 0.0038 g m^−1^·s^−1^·Pa^−1^. Antimicrobial activity tests demonstrated inhibition zones of 11.52 mm for *S. aureus* and 9.31 mm for *E. coli*, attributed to the synergistic effects of Qr's phenolic compounds disrupting bacterial cell membranes and ZnO NPs generating ROS. The antioxidant activity, measured by DPPH free radical scavenging, reached 20 %. Demonstrating strong potential, the nanocomposite film provides an eco-friendly approach to food preservation. This film is especially suitable for low- and intermediate-moisture foods because wheat gluten inherently exhibits excellent oxygen and carbon dioxide barrier properties at low relative humidity. The addition of hydrophobic Qr and ZnONPs further reduces moisture uptake and solubility, making the material ideal for products where moisture sensitivity must be controlled. The reduced WVP and improved mechanical integrity at these optimized conditions indicate the film's suitability for extending the shelf life of foods such as dried fruits, baked snacks, and semi-moist goods. Future research should focus on optimizing nanoliposome incorporation, scaling up production, and evaluating long-term stability to support its commercial application (Bakeshlou et al., 2024a).

### 5.7 Packaging for general food application

**Fig. 14:**
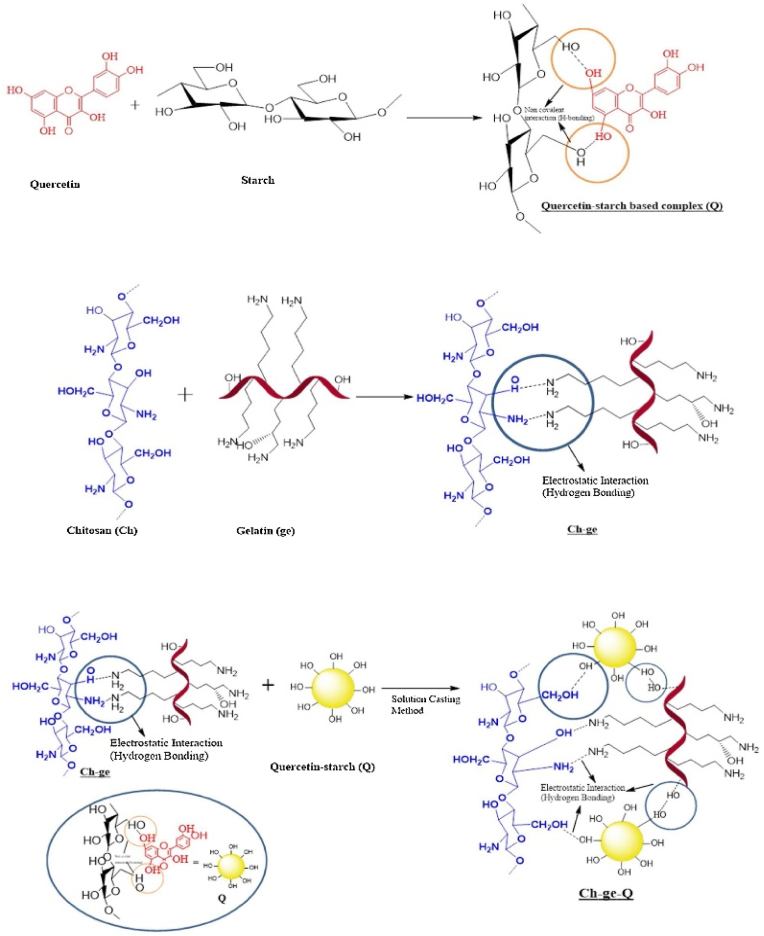
**Preparation of chitosan-gelatin films incorporating a quercetin-starch complex. Reprinted with permission from ref** (Yadav et al., 2020). **copywriter (2020) Elsevier**.

In 2020, Yadav et al. formulated environmentally friendly CS-gelatin (Ch-ge) films infused with Qr-starch for food packaging applications using a manual, batch-based solution casting technique on a small laboratory scale (Fig. 14). In CS film, the TS was 10.54 ± 0.0565 MPa, which increased to 17.11 ± 0.3464 MPa in the Ch-ge-Qr film, while the EAB declined from 11.04 ± 0.0565 % to 5.10 ± 0.3162 %, indicating enhanced mechanical strength with reduced flexibility. Barrier properties improved, with WVP decreasing from 10.12 × 10^−8^ g m^−1^ s^−1^ Pa^−1^ (CS film) to 7.57 × 10^−8^ g m^−1^ s^−1^ Pa^−1^ (Ch-ge-Qr film) and oxygen permeability (OP) reducing from 7.0347 × 10^−6^ cc/m^24^ h atm to 3.582 × 10^−6^ cc/m^24^ h atm. The films exhibited high UV absorption in the 200–400 nm range and extended absorption in the 400–500 nm range, confirming their UV protection ability. Due to Qr's ability to disrupt bacterial cell membranes and inhibit biofilm formation, *E. coli* and *B. subtilis* exhibited clear inhibition zones measuring 25 mm and 24 mm, respectively. Antioxidant activity was significantly enhanced, with DPPH and ABTS scavenging activities reaching 81.45 % and 72.2 %, respectively, at 1 mg/mL concentration. By integrating CS and gelatin, both recognized food-safe biopolymers, with Qr these films demonstrate potent antimicrobial efficacy, superior barrier attributes, and reinforced structural integrity. Their optimal flexibility coupled with minimal oxygen and moisture transmission rates renders them suitable for packaging sensitive food products such as cheese, baked items, ready-to-eat meals, and fresh produce, thereby offering protection against microbial spoilage, oxidative deterioration, and ultraviolet radiation. The study offers promising insights into CS-gelatin-quercetin (Ch-ge-Q) films, yet key gaps remain. Long-term stability, aging, and shelf-life under real food packaging conditions were not evaluated. The solution casting method lacks scalability and economic feasibility assessments for industrial applications. No real-food testing, migration studies, or sensory evaluations were conducted, limiting practical relevance. Despite claims of biodegradability, degradation behavior under compost, landfill, or marine conditions remains untested. Antimicrobial activity was restricted to *E. coli* and *B. subtilis*, warranting broader-spectrum testing. Safety aspects like cytotoxicity and allergenicity were overlooked, and mechanical/barrier properties were only studied in controlled settings. The fixed composition also calls for optimization. Future work should explore real-food trials, scalable manufacturing, expanded antimicrobial testing, environmental impact assessments, and regulatory safety compliance. Incorporating multifunctional bioactives, smart sensors, and consumer acceptance studies will be vital for translating these films into viable, sustainable packaging solutions (Yadav et al., 2020).

López de Dicastillo et al. (2021) synthesized a biodegradable trilayer film by embedding electrospun PCL nanofibers loaded with Qr and CNC between PLA layers. Fabricated at lab scale using manual electrospinning and lamination, the process lacks industrial scalability. Although industrial methods exist, further optimization is needed for continuous, high-throughput production. The mechanical properties improved, with TS increasing to 41.1 MPa and EAB reaching 3.9 %, indicating enhanced structural integrity. Barrier properties, evaluated at different relative humidities, showed no significant improvement compared to PLA, with WVP values of 5.5 × 10^−15^ kg m/m^2^ s Pa at 50 % RH and 3.5 × 10^−15^ kg m/m^2^ s Pa at 90 % RH, suggesting limited resistance to moisture transfer. Thermal characterization revealed a crystallinity increase of 19.1 %, with a glass transition temperature (T_g_) of 61.1 °C, indicating improved thermal stability. Antioxidant analysis demonstrated significant Qr release, reaching 0.82 mg/L in 50 % ethanol and 1.02 mg/L in 95 % ethanol, with CNC enhancing release kinetics, suggesting prolonged oxidative protection. The multilayer PLA-PCL-CNC composite film, formulated from biocompatible and biodegradable polymers, exhibits enhanced TS and thermal stability. Although the WVTR remains largely unaltered, the film demonstrates efficacious antioxidant activity via the sustained and controlled release of Qr, conferring oxidative protection across diverse food matrices, including those with intermediate moisture and lipid contents. These attributes render the material highly suitable for advanced packaging applications involving dairy, meat products, and ready-to-eat meals, where preservation of physicochemical integrity and inhibition of oxidative degradation are paramount. While biodegradable multilayer packaging holds strong potential, several limitations need resolution for practical deployment. Issues like poor long-term stability, weak bonding between layers, and challenges in scaling up fabrication methods such as electrospinning affect functionality and industrial relevance. Limited research on the migration of active compounds also raises food safety concerns, while the environmental impact of embedded nanomaterials remains underexplored. Moreover, the use of a single functional agent narrows the scope of application. Moving forward, research should emphasize scalable manufacturing, improved layer adhesion, in-depth safety and degradation assessments, and the integration of multiple active or intelligent features to enhance performance and consumer assurance (López de Dicastillo et al., 2021).

In 2021, Łopusiewicz et al. developed bioactive PBS films modified with Qr for food packaging applications. The PBS-Qr films were prepared on a small laboratory scale using the solvent casting method. The incorporation of Qr led to changes in the mechanical properties of the films, with TS declining from 11.80 ± 2.20 MPa in neat PBS to 8.40 ± 2.50 MPa in PBS-Qr0.50, and EAB dropping from 155.00 ± 32.10 % to 81.00 ± 9.20 %, indicating decreased flexibility. Barrier properties showed no significant change in WVTR, which remained around 60.27 ± 5.08 g/m^2^·day, suggesting that Qr did not enhance moisture resistance. This stable barrier behavior across different formulations, combined with moderate mechanical performance, makes PBS-Qr films suitable for general food packaging, particularly for products not requiring strict moisture control but benefiting from functional bioactivity. Thermal analysis via DSC and TGA revealed no major impact on PBS's thermal transitions, with melting and crystallization temperatures remaining stable at 84 °C and 42 °C, respectively. The antimicrobial activity of PBS-Qr0.50 films was moderate, reducing *E. coli* to 2.30 × 10^2^ ± 0.60 CFU/mL and *S. aureus* to 1.10 × 10^2^ ± 0.36 CFU/mL, attributed to Qr's ability to disrupt bacterial membranes and inhibit nucleic acid synthesis. Such antimicrobial efficacy, though moderate, is beneficial for general food packaging to extend shelf life and reduce microbial contamination across a broad range of food types. Antioxidant activity was significantly enhanced, with DPPH scavenging reaching 80.90 ± 0.07 % and ABTS scavenging at 99.07 ± 0.28 %, demonstrating strong free radical stabilization by Qr. These high antioxidant properties contribute to the protection of foods susceptible to oxidative spoilage, further supporting the material's relevance for general packaging applications. Migration tests showed Qr release was highest in 96 % ethanol (0.25 ± 0.01 mg/L), indicating its suitability for fatty food applications. Thus, PBS-Qr films exhibit multifunctionality, combining biodegradability with bioactivity, making them broadly suitable for general food applications where moderate barrier, antimicrobial, and antioxidant functions are valued. There is a notable lack of research on incorporating Qr into poly(butylene succinate) (PBS) films for active food packaging, despite PBS's growing relevance as a biodegradable polymer. Current studies offer limited insight into the long-term stability of these films under real storage conditions. Future research should focus on real-world packaging applications, broaden antimicrobial and antioxidant evaluations, assess migration and toxicity across diverse food systems, enhance mechanical properties through material modifications, evaluate environmental and economic feasibility, and explore other natural bioactive compounds to improve overall functionality (Łopusiewicz et al., 2021).

Ezati and Rhim et al. fabricated Qr-loaded biopolymer films from CMC, gelatin, and PLA using a small-scale, manually operated casting technique in 2021. Polymers were dissolved in suitable solvents with plasticizers, blended with Qr in ethanol/Tween 80, and cast onto Teflon sheets to dry under ambient conditions, reflecting an experimental, non-commercial approach. Qr was more compatible with CMC than gelatin and PLA, leading to improved mechanical properties in CMC films, with TS increasing from 34.3 to 42.8 MPa, EAB from 19.9 % to 25.1 %, and EM from 1.14 to 1.23 GPa. However, gelatin and PLA films exhibited decreased TS and EM due to poor compatibility with Qr. These variable mechanical performances across polymer matrices suggest the potential of tailoring the films for different general food packaging needs, ranging from rigid to flexible applications. The UV-blocking ability was significantly enhanced, with UV transmittance at 280 nm (T_280_) reduced to 0.4 %, 0.1 %, and 2.6 % for CMC, gelatin, and PLA films, respectively, while transparency at 660 nm (T_660_) decreased to 51.1 %, 70.7 %, and 43.1 %. This UV shielding is especially important for protecting various general food products from light-induced spoilage, nutrient loss, or discoloration, supporting broad application. WVP slightly decreased for CMC/Qr films (1.6 × 10^−9^ g/m·s·Pa) but increased for gelatin/Qr (1.04 × 10^−9^) and PLA/Qr (0.2 × 10^−9^) films. Antimicrobial activity was observed in CMC/Qr and gelatin/Qr films, significantly inhibiting *Listeria monocytogenes* and *Escherichia coli*, reducing bacterial viability by 2–3 log CFU/mL due to Qr-induced bacterial membrane disruption. The antimicrobial activity further supports their general application for enhancing food safety across multiple food categories, especially where contamination risk is present. Antioxidant activity was also notable, with radical scavenging rates of 68.4 %, 100 %, and 42 % for CMC, gelatin, and PLA films, respectively, in the ABTS assay. The release of Qr was highest in 50 % ethanol at 25 °C, with the fastest release observed in gelatin/Qr films. The films were found to be biocompatible and environmentally friendly, making them suitable for active food packaging applications. The combination of antioxidant and antimicrobial functionality, along with tunable mechanical and barrier properties, reinforces their utility as versatile, general-purpose active packaging materials. However, future research is needed to optimize release kinetics and improve industrial scalability (Ezati and Rhim, 2021).

Wang et al. (2024) created sustainable, active food packaging films by integrating quercetin-functionalized layered clay (QUE-LDHs) into CS and PVA matrices through solution casting. The QUE-LDHs/CS/PVA nanocomposite films were produced at a laboratory scale using solution casting in PTFE molds. This hands-on, batch-oriented technique is commonly used in lab settings and lacks the involvement of industrial-scale machinery. The QUE-LDHs/CS/PVA nanocomposite films exhibited enhanced mechanical properties, with a maximum TS of 58.9 MPa at 3 wt % QUE-LDHs, representing a 40.9 % increase over the CS/PVA matrix. The films demonstrated 100 % UV absorption at 280 nm while maintaining visible light transparency. Thermal analysis revealed an initial decomposition temperature (T-5 %) increase to 101.6 °C at 5 wt% QUE-LDHs, but thermal degradation accelerated at higher temperatures. The films showed 95.5 % antibacterial activity against *E. coli*, resulting from the combined action of quercetin's polyphenol structure and copper ions breaking down microbial biofilms. Antioxidant activity, measured via DPPH radical scavenging, reached 58.9 %, rather than 78.3 % as previously stated. This nanocomposite film is well-suited for general food packaging, offering a balanced combination of high TS, UV protection, antimicrobial and antioxidant properties, and visible light transparency. These features protect various foods from microbial spoilage, oxidative degradation, and photodamage, while allowing product visibility, important for categories such as nuts, dry fruits, and snacks. The research concludes that QUE-LDHs/CS/PVA nanocomposites offer improved mechanical, barrier, and antimicrobial properties, making them a promising material for active food packaging. Future studies should explore the scalability, biodegradability, and real-world application of QUE-LDHs/CS/PVA nanocomposites in food packaging (Wang et al., 2024).

## 6 Safety considerations and environmental effects

Qr, classified as a GRAS-designated edible compound, exhibits minimal safety concerns for its incorporation into food packaging films or coatings. Furthermore, it is extensively utilized as a nutraceutical supplement in several countries, with administered doses ranging from approximately 200 to 1200 mg. In the context of functional foods, the prescribed intake per serving is typically within the range of 10–125 mg (Harwood et al., 2007), (Ulusoy and Sanlier, 2020). However, in vitro studies have indicated that Qr may pose certain health concerns under specific conditions. Its oxidative metabolism leads to the formation of quinone derivatives, which exhibit high reactivity toward thiol-containing biomolecules such as glutathione, resulting in the formation of complexes that are not considered physiologically favorable (Awad et al., 2002). Additionally, some studies have suggested that quinone derivatives of Qr may exhibit genotoxic effects against bacterial strains (Duarte Silva et al., 2000). Despite these relatively minor concerns, Qr remains highly advantageous for human health and is deemed safe for applications in food packaging. Furthermore, no adverse environmental effects associated with its use in food systems have been reported to date.

Although no significant environmental adverse effects have been documented in food system applications, emerging research indicates potential complications regarding biodegradability and compostability when Qr and flavonoids are incorporated as functional additives in biodegradable polymer matrices. Bonnenfant et al. (2023) reported the antimicrobial effects of Qr and gallic acid on PHBV-degrading bacterial strains, particularly *Streptomyces exfoliates,* which showed significant inhibition of microbial proliferation and consequent prolongation of the biodegradation phase under controlled experimental conditions. Complete mineralization of Qr-containing PHBV films was achieved within standardized rates during the linear biodegradation phase and delayed compost initiation relative to control PHBV samples (Bonnenfant et al., 2023). These results indicate that Qr's antimicrobial properties, while enhancing polymer functionality and maintaining overall compostability, may temporarily compromise microbial efficiency in waste degradation systems, necessitating careful consideration in sustainable packaging design strategies.

## 7 Conclusion

In conclusion, Qr-based nanocomposite films epitomize a paradigm shift in food packaging technologies, addressing pressing challenges in food preservation, safety, and environmental sustainability. As a bioactive flavonoid, Qr exhibits unparalleled antimicrobial and antioxidant efficacy, underpinned by its unique molecular architecture characterized by five hydroxyl groups and a conjugated system. This structural motif not only enhances radical scavenging kinetics but also disrupts microbial membranes and biofilm formation, outperforming analogous flavonoids such as catechin and rutin in mitigating oxidative degradation and pathogen proliferation. The incorporation of Qr into biopolymeric matrices, ranging from CS and PVA to advanced nanocomposites, confers remarkable mechanical robustness, superior barrier properties, and enhanced thermal stability. These attributes are critical for preserving the integrity of perishable commodities, including fruits, vegetables, and meat, while extending shelf life by up to 50%. Furthermore, Qr-functionalized films demonstrate exceptional UV-shielding capabilities, mitigating photodegradation in light-sensitive foods, and align with global sustainability goals through their inherent biodegradability and reduced ecological footprint. Among fabrication techniques, solution casting is widely used at laboratory scale for developing nanocomposite films due to its conceptual simplicity and potential for homogeneous dispersion of nanofillers within polymeric matrices. However, its suitability for industrial-scale production is debatable and limited by significant challenges. The process involves solvent-based steps requiring large solvent volumes, which raises environmental, safety, and cost concerns related to solvent recovery and emissions. Moreover, the drying stage is highly time-consuming (often several hours to days), energy-intensive, and difficult to control uniformly at a large scale, leading to batch-to-batch inconsistencies in film thickness and nanofiller dispersion. The maximum achievable film dimensions during casting are also limited, constraining throughput and scalability. Additionally, the risk of nanofiller agglomeration grows with scale-up, adversely impacting the functional and mechanical performance of the final films. These limitations reduce the industrial attractiveness of solution casting despite its prevalent use in research settings. Alternative scalable fabrication methods such as extrusion (single or twin-screw), compression molding, and co-extrusion provide continuous processing options with reduced solvent use and improved throughput, though these also come with challenges like higher equipment costs and sometimes reduced control over nanofiller dispersion. Future efforts should focus on optimizing these scalable techniques and incorporating advanced mixing and dispersion strategies to balance process efficiency with nanocomposite film quality. Emphasizing sustainable solvent management or solvent-free methods will be crucial for industrial adoption of bioactive nanocomposite packaging materials like Qr-based films.

Future research must prioritize optimizing film formulations to address challenges such as moisture sensitivity, mechanical flexibility, and long-term stability under real-world conditions. The integration of smart and active packaging systems, including pH-responsive indicators, real-time freshness sensors, temperature-sensitive labels, and controlled-release antimicrobial systems, could further enhance functionality. Additionally, the incorporation of biodegradable sensors and IoT-enabled tracking could revolutionize supply chain transparency, enabling dynamic quality control from production to consumption. Self-healing materials research into Qr-based edible coatings and multi-layer nanocomposites could optimize performance while minimizing material use. Advanced characterization techniques, including molecular dynamics simulations and in situ spectroscopic analysis, could elucidate structure-property relationships to guide material design. Interdisciplinary collaboration, spanning material science, food technology, and environmental engineering, is essential to overcome scalability and cost barriers while ensuring compliance with regulatory standards. Moreover, circular economy strategies such as agricultural waste upcycling and enzyme-assisted recycling should be prioritized to close the material loop. By leveraging these innovations, Qr-based nanocomposites are poised to revolutionize food packaging, offering a sustainable, intelligent, and high-performance alternative to conventional plastics. This transformative approach not only mitigates food waste but also supports a circular economy, reinforcing its pivotal role in the future of global food security and environmental stewardship.

## CRediT authorship contribution statement

**Sakshi Jasrotia:** Conceptualization, Methodology, Data curation, Writing – original draft, Visualization. **Sonali Gupta:** Methodology, Writing – original draft, Visualization. **Manas Laxman Kudipady:** Data curation, Writing – original draft. **Yashoda Malgar Puttaiahgowda:** Writing-Review Editing, Supervision, and Project administration.

## Ethical approval

This study did not involve the use of animals and complies with ethical guidelines.

## Declaration of competing interests

The authors have no conflict of interest to declare.

## Data Availability

Data will be made available on request.
